# Interleukin-17A/F1 Deficiency Reduces Antimicrobial Gene Expression and Contributes to Microbiome Alterations in Intestines of Japanese medaka (*Oryzias latipes*)

**DOI:** 10.3389/fimmu.2020.00425

**Published:** 2020-03-17

**Authors:** Yo Okamura, Natsuki Morimoto, Daisuke Ikeda, Nanami Mizusawa, Shugo Watabe, Hiroshi Miyanishi, Yuichi Saeki, Haruko Takeyama, Takashi Aoki, Masato Kinoshita, Tomoya Kono, Masahiro Sakai, Jun-ichi Hikima

**Affiliations:** ^1^Interdisciplinary Graduate School of Agriculture and Engineering, University of Miyazaki, Miyazaki, Japan; ^2^School of Marine Biosciences, Kitasato University, Sagamihara, Japan; ^3^Department of Marine Biology and Environmental Science, Faculty of Agriculture, University of Miyazaki, Miyazaki, Japan; ^4^Department of Biochemistry and Applied Bioscience, Faculty of Agriculture, University of Miyazaki, Miyazaki, Japan; ^5^Department of Life Science and Medical Bioscience, School of Advanced Science and Engineering, Waseda University, Tokyo, Japan; ^6^Integrated Institute for Regulatory Science, Research Organization for Nao and Life Innovation, Waseda University, Tokyo, Japan; ^7^Division of Applied Bioscience, Graduate School of Agriculture, Kyoto University, Kyoto, Japan

**Keywords:** genome editing, interleukin-17A/F1, Japanese medaka, *Oryzias latipes*, transcriptome analysis, metagenomics, teleosts

## Abstract

In mammals, interleukin (IL)-17A and F are hallmark inflammatory cytokines that play key roles in protection against infection and intestinal mucosal immunity. In the gastrointestinal tract (GI), the induction of antimicrobial peptide (AMP) production via Paneth cells is a fundamental role of IL-17A and F in maintaining homeostasis of the GI microbiome and health. Although mammalian IL-17A and F homologs (referred to as IL-17A/F1-3) have been identified in several fish species, their function in the intestine is poorly understood. Additionally, the fish intestine lacks Paneth cells, and its GI structure is very different from that of mammals. Therefore, the GI microbiome modulatory mechanism via IL-17A/F genes has not been fully elucidated. In this study, Japanese medaka (*Oryzias latipes*) were used as a teleost model, and IL-17A/F1-knockout (IL-17A/F1-KO) medaka were established using the CRISPR/Cas9 genome editing technique. Furthermore, two IL-17A/F1-deficient medaka strains were generated, including one strain containing a 7-bp deletion (-7) and another with an 11-bp addition (+11). After establishing F2 homozygous KO medaka, transcriptome analysis (RNA-seq) was conducted to elucidate IL-17A/F1-dependent gene induction in the intestine. Results of RNA-seq and real-time PCR (qPCR) demonstrated down-regulation of immune-related genes, including interleukin-1β (*IL-1*β), complement 1q subunit C (*C1qc*), transferrin a (*Tfa*), and G-type lysozyme (*LyzG*), in IL-17A/F1-KO medaka. Interestingly, protein and lipid digestive enzyme genes, including phospholipase A2, group IB (*pla2g1b*), and elastase-1-like (*CELA1*), were also downregulated in the intestines of IL-17A/F1-KO medaka. Furthermore, to reveal the influence of these downregulated genes on the gut microbiome in IL-17A/F1-KO, 16S rRNA-based metagenomic sequencing analysis was conducted to analyze the microbiome constitution. Under a non-exposed state, the intestinal microbiome of IL-17A/F1-KO medaka differed at the phylum level from wild-type, with significantly higher levels of Verrucomicrobia and Planctomycetes. Additionally, at the operational taxonomic unit (OTU) level of the human and fish pathogens, the Enterobacteriaceae *Plesiomonas shigelloides* was the dominant species in IL-17A/F1-KO medaka. These findings suggest that IL-17A/F1 is involved in the maintenance of a healthy gut microbiome.

## Introduction

In mammals, the relationship between host mucosal immunity in the gastrointestinal tract (GI) and a good microbiome environment has received increased attention with regards to promoting body health (1). The human microbiome is comprised of different types of microorganisms, including bacteria (prokaryotes), archaea, fungi, protists, and viruses, which inhabit the GI, skin, oral cavity, urogenital organ, and nasal fossa, forming different and uniquely diverse microbiomes (2). In the GI, the number of inhabiting microorganisms has been estimated to exceed 10^14^, with bacteria representing a major player in the GI microbiome (3, 4). GI microbiomes are modulated by various host biochemical factors, such as pH in the stomach, antimicrobial peptides (AMPs) in the small intestines, and mucin (MUC) in the colon (4). Failure to modulate this balance can lead to deterioration of enteritis pathophysiology, such as Crohn's disease and ulcerative colitis (5).

Interleukin 17 (IL-17) is a hallmark inflammatory cytokine produced by the T helper 17 (Th17) subset of CD4+ T cells that plays key roles in protection against infection and intestinal mucosal immunity and is also a critical microbiome regulator in mammals (6). Th17 subset of CD4+ T cells have been reported to accumulate at a high density in the mammalian intestinal tract, and it is known that both transforming growth factor (TGF-β) and IL-6 are essential for Th17 differentiation (7). Furthermore, segmented filamentous bacteria (SFB), belonging to the genus of *Clostridium*, play important roles in Th17 differentiation (8, 9), where SFB antigen presentation by resident dendritic cells in the intestinal tract has been suggested as a key step in the process (10). Studies on the relationship between the Th17-mediated intestinal immune system and gut microbiome have increased in recent years. It has recently been revealed that IL-17 is produced from other lymphocytes, γδ-T cells, natural killer T (NKT) cells, innate lymphoid cells (ILC3), neutrophils, mast cells, and macrophages (11). Currently, six family members, namely IL-17A, B, C, D, E, and F, have been identified in mammals (12). Among them, IL-17A and F are known as key inflammatory cytokines that modulate the microbiome. IL-17A and F share the highest homology among IL-17 family members, and their gene loci are adjacent to each other on the same chromosome (13). These two IL-17s can bind both the IL-17 receptor A (RA) and receptor C (RC) heterodimer complex. However, IL-17F has a decreased ability to induce inflammation compared to IL-17A, and the reason why remains unknown. Moreover, differences in the roles of IL-17A and IL-17F in pathogenesis have yet to be elucidated (12). Nevertheless, IL-17A and F are known to induce pro-inflammatory cytokines such as IL-1β, IL-6, and TNF-α, the chemokines CXCL1, CXCL8, and CCL20, and AMPs, including defensin and calprotectin (11, 14). The mammalian intestinal tract, particularly the small intestine, exhibits a unique AMP production mechanism via Paneth cells. Paneth cells are neutrophil-like, intestinal epithelial cells that contain granules and are localized in crypts of villus tissue, with an ability to produce AMPs such as phospholipase A2, lysozyme, and defensin in the intestine (15). Of these, the human alpha-defensin (HD5) homolog gene in mice, cryptdin (Crp)—a representative AMP secreted from Paneth cells—potently kills pathogens, yet it shows almost no anti-bactericidal activity against commensal bacteria (16). Moreover, Paneth cells transmit signals to adjacent intestinal epithelial stem cells via Wnt and Notch signaling and play an important role in determining the differentiation pathway of each epithelial cell (15). Thus, AMP exhibits anti-bactericidal activity with some selectivity. Therefore, IL-17A and F are considered critical for pathogen elimination and maintenance of the gut microbiome via the intestinal immune system. By contrast, in fish, the relationship between IL-17 and other immune systems and the gut microbiome has hardly been clarified. Therefore, we focused on the importance of the regulatory function of IL-17 on the gut microbiome in maintaining fish health.

In teleosts, the mammalian IL-17A and F homologs, referred to as IL-17A/F1-3, have been identified in many fish species such as zebrafish (*Danio rerio*), Japanese medaka (*Oryzias latipes*), Japanese puffer (*Takifugu rubripes*), channel catfish (*Ictalurus punctatus*), European seabass (*Dicentrarchus labrax*), rainbow trout (*Oncorhynchus mykiss*), and Atlantic salmon (*Salmo salar*) (17–21). Among the three types of IL-17A/F genes, IL-17A/F1 and IL-17A/F2 are adjacently located on the same chromosome, similar to that of mammalian IL-17A and F, but share low sequence identity. IL-17A/F3 is located on a different chromosome from that of IL-17A/F1 and 2 and shows higher amino acid sequence identity and similarity to IL-17A/F1 than to IL-17A/F2. Moreover, phylogenetic analysis determined that IL-17A/F3 forms a cluster that is closer to IL-17A/F1 than to IL-17A/F2 (20). It has been reported that expression levels of IL-17A/F1, 2, and 3 genes fluctuate at the mRNA level during bacterial infection in multiple fish species. *Yersinia ruckeri* up-regulates salmonid IL-17A/F1, 2, and 3 in the spleen (22), whereas *Aeromonas hydrophila* up-regulates large yellow croaker counterparts in the head, kidney, and gill (23). However, details regarding the mechanism of fish IL-17A/F gene regulation of the intestinal tract microbiome are unknown. Furthermore, in the teleost gut, Paneth cells have not been found. In addition, the pH of the teleost GI is closer to neutral compared with that of mammals (24). Based on these differences in the GI physiological structure, we hypothesized that the aquatic environment drives different mechanisms to control the gut microbiome in teleosts compared with land mammals.

In this study, we aimed to understand the role of IL-17A and F in the intestines of teleosts by constructing two IL-17A/F1-mutated strains of Japanese medaka (*O. latipes*) [IL-17A/F1-knockout(KO)-medaka] using CRISPR-Cas9 genome editing technology. After establishing the mutant strains, transcriptome analysis (RNA-seq) was conducted to investigate IL-17A/F1-dependent gene regulation. Additionally, 16S metagenome analysis of intestinal content was performed. This is the first report using IL-17 gene-mutated strains to extensively analyze the role of IL-17 in the intestines of teleosts.

## Materials and Methods

### Medaka

The Cab strain of Japanese medaka was maintained in several transparent plastic tanks with a water circulating system (26°C) under a 14-h light and 10-h dark cycle. In all experiments, fish that were 3–4 months old and weighing 200–300 mg were used. All animal experiments were conducted according to the relevant national (Act on Welfare and Management of Animals, Ministry of the Environment, Japan) and international guidelines. Ethics approval from the local IACUC was not sought as this law does not mandate protection of fish.

### Establishment of IL-17A/F1-Deficient Medaka Strains

**Figure 1:**
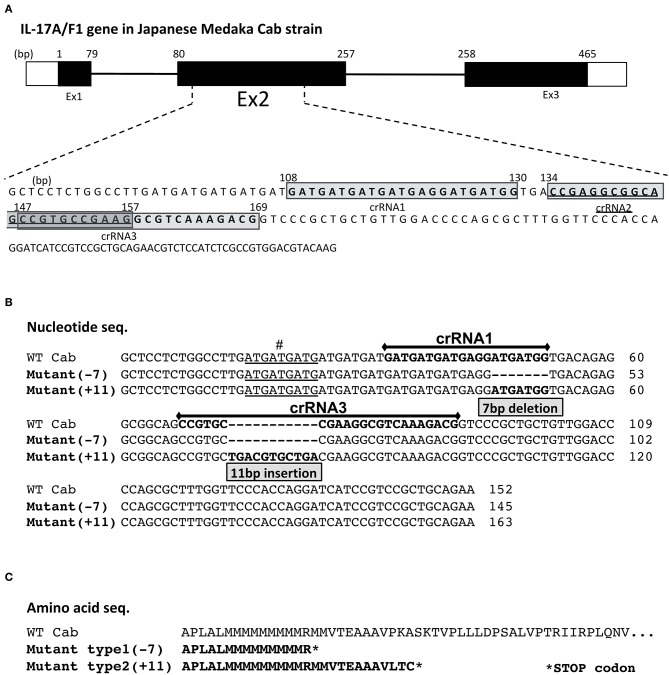
crRNA regions and mutated sequences in the medaka IL-17A/F1 gene. **(A)** Three crRNA regions in the medaka IL-17A/F1 gene. Three crRNAs were designed in exon 2 of the IL-17A/F1 gene. The mutated nucleotide **(B)** and amino acid sequences **(C)** of WT and two KO strains. The mutated regions in F0 founder and F2 homo individuals were confirmed by sequencing. The nine underlined nucleotides “#” indicate different nucleotide sequences of the IL-17A/F1 gene in the medaka Cab strain compared with that of the Hd-rR strain and GenBank-deposited sequence (acc. no. NM_001204785). In **(C)**, both mutant strains contained premature stop codons in the amino acid sequences due to frameshift mutations.

Approximately 0.5 nL of a solution containing crRNA (25 ng/μL), tracrRNA (40 ng/μL), and Cas9 mRNA (100 ng/μL) was injected into one- to two-cell stage embryos with a manipulator (Narishige, Tokyo, Japan). All three crRNAs were designed in exon 2 of the medaka IL-17A/F1 gene (Figure 1A). Prior to designing crRNAs, the medaka Cab IL-17A/F1 mRNA nucleotide sequence was deposited in the GenBank database under accession number LC501388. The three crRNA sequences are listed in Supplementary Table 1. After injection into the embryo, genomic DNA was extracted from randomly selected embryos to confirm gene editing efficiency using the heteroduplex mobility assay (HMA; Supplementary Figure 1). Filial generation 0 (F0) fish, grown from the injected fertilized eggs, were interbred with wild-type medaka (Cab strain) to produce F1 heterozygotes. Among the F1 fish, males and females carrying an identical mutation were mated (in-crossed) to obtain homozygous progeny and/or a mutant line. For detection of the mutated locus in the genome, HMA was performed. Additionally, to confirm crRNA efficiency, primer sets amplifying specific regions, including the crRNA identical regions, were used; locations are listed in Supplementary Table 1. Briefly, genomic DNA was prepared from the embryo or an excised fin by dissolving in 20 μL of a solution containing 0.2 mM EDTA and 25 mM NaOH and incubating at 95°C for 20 min. After incubation, samples were neutralized by the same volume of 40 mM Tris/HCl (pH 8.0). Before collecting the tail fins, the medaka were anesthetized using MS-222 (Sigma-Aldrich, St. Louis, MO, USA). Using this genomic DNA-containing solution as a template, PCR was carried out using KOD FX Neo (Toyobo, Osaka, Japan). PCR amplification was carried out in a total volume of 10 μL containing 5 μL PCR buffer, 2 μL dNTP, 0.2 μL KOD FX Neo, 1 μL gDNA-containing solution, 0.2 μL each of the forward (Ol_IL-17A/F1_F1) and reverse primers (Ol_IL-17A/F1_R1) at a final concentration of 5 pmol, and 1.4 μL distilled water. The PCR program was initially run at 94°C for 2 min, followed by 37 cycles of 94°C for 30 s, 62°C for 30 s, and 72°C for 20 s. The amplicon products were separated by 12% PAGE to compare their migration patterns.

### RNA Extraction and cDNA Synthesis for Real-Time PCR (qPCR) Analysis and Next-Generation Sequencing

For qPCR analysis, total RNA was extracted from whole intestines of adult Japanese medaka Cab strains using an RNAiso Plus Kit (TaKaRa Bio, Kusatsu, Japan) according to manufacturer's instructions. Total RNA from each medaka was extracted separately (not normalized). Total RNA was quantified with a NanoDrop spectrophotometer (Thermo Fisher Scientific, Waltham, MA, USA). RNA purity was measured by the OD_260_/OD_280_ ratio, with a minimum ratio of 1.8 as the quality cut-off value. cDNA was synthesized using 500 ng of total RNA extracted from each sample with the ReverTra Ace qPCR RT Master Mix with gDNA Remover (Toyobo) according to manufacturer's instructions. For RNA-seq, total RNA from five individuals was equally pooled, and KAPA Stranded RNA-Seq Library Preparation Kit (KAPA Biosystems, Wilmington, MA, USA) was used to construct cDNA libraries according to manufacturer's instructions. cDNA libraries were sequenced using the MiSeq Reagent Kit v3 (150 Cycles; Illumina, San Diego, CA, USA).

### Mapping of Sequence Reads, Differential Expression Analysis, and Gene Enrichment Analysis

Processed reads were submitted to the DDBJ Sequence Read Archive (DRA) under accession number DRA008715. Subsequently, the collected reads were mapped to the annotated medaka Hd-rR reference genome (release 85; http://www.ensembl.org/index.html) with the TopHat program (25) and further analyzed by the Cufflinks-Cuffdiff program (26, 27). Transcription expression values were estimated as fragments per kilobase of exon length per million reads (FPKM), and transcripts with a *q* < 0.05 were considered significantly differentially expressed (26). To normalize individual differences, the RNA-seq samples were pooled from individual fish that were maintained at a constant temperature, and RNA-seq was not replicated. Therefore, the statistical analysis was not reliable, although the Cuffdiff program can calculate *q*-values without biological replicates. Thus, we focused on genes whose expression levels differed substantially (4-fold or higher) between the WT and IL-17A/F1-KO medaka intestinal tissues. Gene enrichment analysis was carried out with the DAVID program (28). Gene ontology (GO) terms in the biological processes (GOTERM_BP_FAT) and the cellular component (GOTERM_CC_FAT) as well as Kyoto Encyclopedia of Genes and Genomes (KEGG) pathways were selected. GO terms were summarized and visualized with the REVIGO program (29).

### Gene Expression Analyses by qPCR

qPCR was used to analyze differences in expression patterns between WT and KO intestinal tissues. We compared IL-17A/F1 (+11) homo-mutant, WT, and medaka exposed with *Edwardsiella piscicida* (immersion in 6.9 × 10^8^ CFU/mL *E. piscicida* for 24 h). Total RNA was extracted from the intestines of medaka (*n* = 5) from each group, and their respective cDNAs were prepared separately as described in section RNA Extraction and cDNA Synthesis for Real-Time PCR (qPCR) Analysis and Next-Generation Sequencing. Target genes for expression analysis were selected based on RNA-seq results, and the primers used are listed in Supplementary Table 1. qPCR amplification was carried out in triplicate in a total volume of 15 μL containing 7.5 μL Brilliant III Ultra-Fast SYBR® Green QPCR Master Mix (Agilent Technologies, Santa Clara, CA, USA), 1.5 μL cDNA, 1.5 μL (5 pmol) each of the forward and reverse primers, and 3 μL distilled water. The qPCR program was run at 95°C for 15 s and 60°C for 30 s, followed by 40 cycles on a CFX connect TM (Bio-Rad Laboratories, Hercules, CA, USA). A melting curve analysis of the amplified products was performed at the end of each cycle to confirm the specificity of amplification. The medaka β*-actin* gene served as an internal control to confirm the quality and quantity of the cDNA. Relative expression ratios were calculated based on the comparative threshold cycle (CT) method (2^−ΔΔCT^ method). Briefly, the target gene and internal control Ct values were determined for each sample, after which the average Ct value of the three replicates was used to calculate expression levels relative to β-actin.

### Sampling Intestinal Contents and Bacterial Genomic DNA Extraction

Before dissection, the surface of medaka bodies was washed three times with PBS containing 0.2% Tween® 20 (molecular biology grade; Promega, Madison, WI, USA) to avoid outer bacterial contamination. After washing, whole intestinal tissues were extracted, and intestinal tracts were filled with PBS and squeezed to extract intestinal contents. From each individual medaka's intestinal content, bacterial genomic DNA was extracted separately (not normalized) using the QIAamp Fast DNA Stool Mini Kit (Qiagen, Hilden, Germany) according to manufacturer's instructions. The concentration of each DNA sample was set to 5 ng/μL using nuclease-free water for the PCR reaction (not pooled).

### DNA Amplification and Sequencing for 16S rRNA Metagenomic Analysis

The V3-V4 region of 16S rRNA was targeted for PCR amplification, and the first PCR was carried out in a total volume of 20 μL containing 10 μL KAPA HiFi HotStart ReadyMix (2X; Nippon genetics, Tokyo, Japan), 8 μL cDNA, and 1 μL (5 pmol) each of the forward (V3V4f) and reverse primers (V3V4r). Primer sequences for the first and second PCR are listed in Supplementary Table 1. The PCR program was run at 94°C for 2 min, followed by 28 cycles of 94°C for 30 s, 55°C for 30 s, and 72°C for 30 s. Before reading sequencing analysis, PCR products were examined for target specificity by 2% agarose gel electrophoresis and purified using AMPure XP (Beckman Coulter, Brea, CA, USA). Using purified DNA, the second PCR was carried out in a total volume of 10 μL containing 1 μL 10X Ex Buffer, 0.8 μL dNTPs (each 2.5 mM), 0.5 μL (10 pmol) each of the forward (2ndF) and reverse primers (2ndR), 2.0 μL purified DNA (purified 1st PCR product, maximum 2 ng/μL), 0.1 μL ExTaq HS (TaKaRa Bio), and 5.1 μL DDW. After amplification, PCR products were purified using AMPure XP, and then purified DNA derived from each individual was used as a library. Before sequencing, library qualities were assessed by Fragment Analyzer and dsDNA 915 Reagent Kit (Advanced Analytical Technologies, Santa Clara, CA, USA). Sequencing was performed under the condition of 2 × 300 bp read lengths using MiSeq (Illumina).

### Data Analysis of 16S rRNA Metagenomic Sequencing

After sequencing, we extracted only those sequences whose beginning sequence reads were an exact match of the primers used by the Fastaq barcode splitter of the Fastx toolkit. The raw paired-end readings were then subjected to a quality control procedure using QIIME 2 (30). Qualified reads were clustered to generate operational taxonomic units (OTUs) at the 97% similarity level, and phylogenetics were estimated using Greengenes OTU (version 13.8). Chimeric sequences were removed using the UCHIME algorithm (version 4.1). The processed 16S sequence data from this study have been deposited at the DRA under accession number DRA008844. For diversity tests and statistical analysis, we executed R codes in RStudio v1.1.442 (31) and employed the below-mentioned packages to analyze the data. Data were imported into R using the package “xlsx.” Observed OTU number and Chao 1 were used to reflect community richness, and diversity was assessed using Shannon indices. In the β-diversity test, we performed weighted UniFrac analysis. For statistical analysis, the abundance of phylum, family, and OTU level between WT and KO was assessed by a Student's *t*-test with subsequent Bonferroni correction. Furthermore, Chao1 and Shannon indices, Kruskal–Wallis test, and Dunn's test with the Benjamini–Hochberg FDR correction using “dunn.test” and “FSA” in R package were used to identify significant differences in the α-diversity indices of the groups. In weighted UniFrac analysis, *PERMANOVA* using “MASS” and “Vegan” in R package were used to identify significant differences in distance between different groups.

### qPCR Quantification of *Plesiomonas shigelloides*

qPCR was used to calculate the plasmid copy number of *P. shigelloides* in each of the WT and KO groups. The *P. shigelloides* genomic DNA fragment (GenBank accession number AJ300545) was amplified via PCR using specific primers from the DNA sample extracted for metagenome analysis. PCR products were cloned into the pTAC-2 vector (BioDynamics, Kumamoto, Japan), and plasmid DNA was extracted using the QIAprep Spin Mini-prep Kit (Qiagen) from transformed *Escherichia coli* (DH5α). Extracted plasmid DNA was diluted from 1.0 × 10^10^ to 1.0 × 10^3^ copies/μL to create a standard curve. qPCR amplification was carried out in triplicate in a total volume of 15 μL containing 7.5 μL Brilliant III Ultra-Fast SYBR® Green QPCR Master Mix, 5.5 μL DNA, and 1.0 μL (5 pmol) each of the forward and reverse primers. The qPCR program was run at 95°C for 10 s and 60°C for 20 s, followed by 40 cycles on a CFX connect TM.

### Medaka Exposure With *E. piscicida*

The Japanese medaka Cab strain was exposed by immersion in fresh water containing *E. piscicida* [E381 strain (former name *E. tarda*) isolated from the Nile tilapia (*Oreochromis niloticus*) kindly gifted by Dr. Tomokazu Takano]. *E. piscicida* was cultured with heart infusion medium (Becton, Dickinson and Company, Franklin Lakes, NJ, USA) at 28°C for 48 h. After 24-h immersion of medaka in freshwater + *E. piscicida*, the water in the tanks was changed to new freshwater without *E. piscicida*. CFU counts in the tank water during bacterial challenge were determined by the plate counting method. The concentration of the *E. piscicida* in the freshwater was confirmed by plate counting methods (i.e., gene expression experiment by qPCR; 6.9 × 10^8^ CFU/mL and metagenomic experiment; 2.1 × 10^8^ CFU/mL). For both experiments, five fish were selected at 0, 24, and 48 h from WT and IL-17A/F1-KO(+11) groups.

## Results

### Establishment of Two IL-17A/F1-KO Medaka Strains

Of the three crRNAs located in exon 2 of IL-17A/F1, crRNA1 and 3 showed a high mutation level (Supplementary Figure 1). After injection into embryos with an RNA mixture containing crRNA, tracRNA, and Cas9 mRNA, two strains of IL-17A/F1-KO medaka were obtained. One strain had a 7-bp deletion (-7) in the region of the crRNA1, and another strain had an 11-bp insertion (+11) in crRNA3 (Figure 1B). For both KO strains, the amino acid sequence of IL-17A/F1 was terminated in the middle of its full-length sequence due to a codon frame shift (Figure 1C). The IL-17A/F1-KO(+11) strain did not show any morphological abnormalities in juvenile and adult fish (Supplementary Figures 2A,B). In F2 homo-mutant individuals, no differences were found in intestinal villus tissue (Supplementary Figure 2C), and no significant changes in body weight were observed between WT and IL-17A/F1-KO medaka (Supplementary Figure 2D).

### Expression of Three IL-17A/F Genes in WT and IL-17A/F1-KO Medaka Intestines

**Figure 2:**
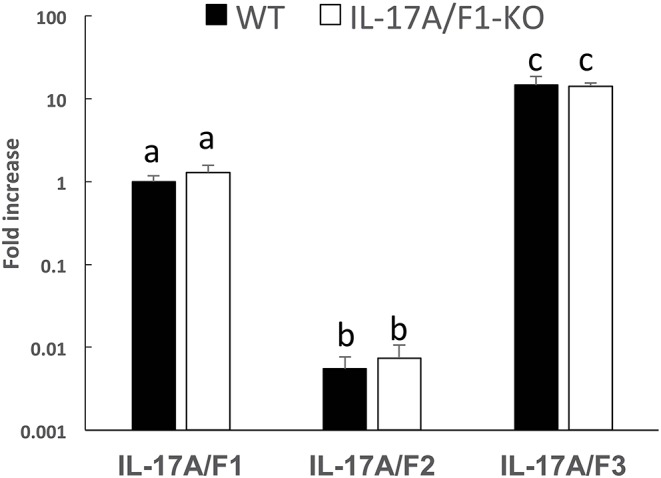
Gene expression analysis of IL-17A/F1, 2, and 3 in WT and KO medaka. No significant changes in the three IL-17A/Fs at the transcriptional level were observed in KO medaka (+11 inserted strain) intestines compared with WT. Comparisons were performed using healthy medaka. Symbols with different letters are significantly different at *p* < 0.05 with Tukey Kramer's multiple comparison test after a one-way ANOVA. Data are presented as mean ± SD; *n* = 5 fish' SD depends on the statistical analyses run.

Of the three IL-17A/F genes in the WT intestine, the expression level of IL-17A/F3 was the highest, followed by IL-17A/F1 and 2, which was statistically significant (Figure 2). Furthermore, no changes were observed in the expression patterns of IL-17A/F1, 2, and 3 in IL-17A/F1-KO(+11) (primer set of IL-17A/F1 for qPCR amplified the extra region of the +11 bp mutation) compared to WT.

### Transcriptome Analysis of WT and IL-17A/F1-KO Medaka Intestines

**Figure 3:**
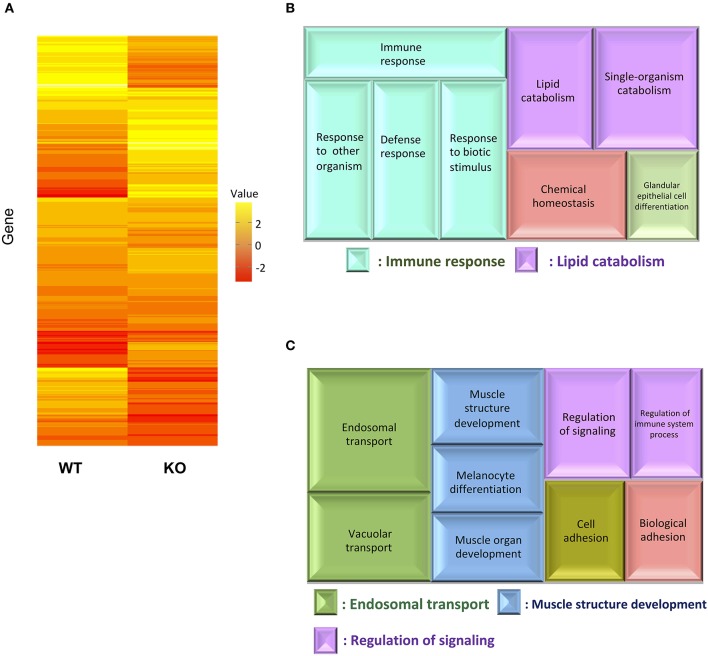
Comparative analyses between WT and IL-17A/F1-KO intestines by RNA-seq. **(A)** Differences in expression patterns of detected genes. Heatmaps of RNA-seq were generated using unsupervised hierarchical clustering with Euclidian distance metric using 863 genes. The input matrix on rows has been converted to –log10 *p*-value. **(B,C)** Treemap view of the summarized lists of GO terms in the biological process category. The size of the color panel reflects the number of relevant genes. Enriched GO terms for genes down-regulated by ≥4-fold in IL-17A/F1-KO (7-bp deletion strain) **(B)** and upregulated by ≥4-fold in KO(-7) **(C)**.

For transcriptome analysis, F2 homo individuals of the IL-17A/F1(-7)-deficient strain were used to compare gene expression in whole intestines with that of the WT. Next-generation sequencing provided the 2993350 and 4443631 raw sequence reads from WT and KO cDNA samples, respectively. RNA-seq revealed that WT and IL-17A/F1-KO medaka had different expression patterns under the non-exposed state (Figure 3A). Furthermore, REVIGO analysis showed down-regulation in several genes that participate in the immune response and lipid catabolism in KO medaka intestines (Figure 3B). Conversely, gene groups related to endosomal transport and muscle structure development were mainly up-regulated (Figure 3C). Table 1 shows a list of genes with a significant down-regulation of ≥4-fold in the IL-17A/F1-KO intestines compared with WT, and includes various immune-related genes, such as AMPs, transferrin a (*Tfa*), and complement 1q subunit C (*C1qc*) located on chromosome 18. In addition to these genes, various lipid and protein digestive enzyme genes, including phospholipase A2, group IB (*pla2g1b)*, bile salt-activated lipase-like (*CEL*), and elastase 1 (*CELA1*), were down-regulated (≥4-fold) in IL-17A/F1-KO intestines compared with WT. Furthermore, Table 2 lists several genes, including craniofacial development protein 2-like (*CFDP2L*), mitogen-activated protein kinase 4 (MAP4K4), and ethanolamine-phosphate phospho-lyase (*ETNPPL*), that were found to be significantly up-regulated (≥4-fold) in IL-17A/F1-KO intestines compared with WT.

**Table 1:** List of genes down-regulated (1/4-fold) in IL-17A/F1-KO medaka intestines.

**Gene_ID**	**Gene name**	**Gene symbol**	FPKM^*1^		***q*-value**	Fold decreases^*2^
			**WT**	**IL-17A/F1 KO**		
ENSORLG00000017611	Cytochrome P450, family 8, subfamily B, polypeptide 2	*cyp8b2*	207.5560	51.5596	0.0085	0.2484
ENSORLG00000006426	Chymotrypsin-like protease CTRL-1	*ctrl*	291.0030	68.0450	0.0085	0.2338
ENSORLG00000019499	Astacin like metallo-protease	–	1448.2400	335.6970	0.0085	0.2318
ENSORLG00000013259	Capicua transcriptional repressor b	*cicb*	21.6388	4.8732	0.0085	0.2252
ENSORLG00000005210	Alpha-type globin	*Gb-alpha1*	224.9320	48.6720	0.0085	0.2164
ENSORLG00000010032	Carboxypeptidase A4	*CPA3*	1747.2800	377.4020	0.0085	0.2160
ENSORLG00000002377	Acyl-coenzyme A thioesterase 5	*Acot5*	102.8930	21.2034	0.0085	0.2061
ENSORLG00000007857	Complement C1q subcomponent subunit C	*C1qc*	154.3540	30.5793	0.0085	0.1981
ENSORLG00000016512	Transferrin	*tfa*	36.5878	7.1960	0.0085	0.1967
ENSORLG00000000239	Astacin like metallo-protease	*c6ast3*	1031.8000	202.8680	0.0085	0.1966
ENSORLG00000008484	STEAP4 metalloreductase	*steap4*	76.5607	15.0486	0.0085	0.1966
ENSORLG00000000965	Carboxypeptidase A2	*cpa4*	569.1470	107.4070	0.0085	0.1887
ENSORLG00000018515	si:dkey-266f7.9	*si:dkey-266f7.9*	110.1450	20.0705	0.0085	0.1822
ENSORLG00000020058	Endonuclease, poly(U) specific	*endou*	353.4440	58.4330	0.0085	0.1653
ENSORLG00000019810	Carboxypeptidase A1	*CPA1*	2222.3400	363.8200	0.0085	0.1637
ENSORLG00000014439	Bile salt-activated lipase	*CEL* (1 of many)	2339.8800	382.6620	0.0085	0.1635
ENSORLG00000003310	Betaine–homocysteine S-methyltransferase 1	*bhmt*	413.2460	64.8265	0.0085	0.1569
ENSORLG00000018361	Phospholipase A2 group IB	*pla2g1b*	284.4890	41.1248	0.0085	0.1446
ENSORLG00000011457	Bactericidal permeability-increasing protein	*LBP2*	112.0850	15.3312	0.0085	0.1368
ENSORLG00000016356	Peptidoglycan recognition protein 2 (pglyrp2)	*pglyrp2*	369.8330	45.1395	0.0085	0.1221
ENSORLG00000000459	Non-specific cytotoxic cell receptor protein 1	*nccrp1*	146.6040	14.0120	0.0085	0.0956
ENSORLG00000017313	3-hydroxy-3-methylglutaryl-coenzyme A reductase	*hmgcra*	89.4073	4.6232	0.0085	0.0517
ENSORLG00000000412	Solute carrier family 12 member 3	*slc12a3*	65.3937	2.2322	0.0085	0.0341
ENSORLG00000003181	NADPH oxidase organizer 1	*noxo1b*	12.6958	0.0000	0.0085	0.0000
ENSORLG00000004187	Complement C1q-like protein 2	*C1qL2*	8.1447	0.0000	0.0085	0.0000
ENSORLG00000014743	Pleckstrin homology domain-containing family B member 1	*PLEKHB1*	7.9812	0.0000	0.0085	0.0000
ENSORLG00000013659	Oviduct-specific glycoprotein	*OVGP1* (1 of many)	104.5850	23.1455	0.0154	0.2213
ENSORLG00000014464	Exoglucanase 1	*CEL* (1 of many)	1254.8900	260.1720	0.0154	0.2073
ENSORLG00000001769	Zinc transporter 8	*slc30a8*	56.8792	7.9022	0.0219	0.1389
ENSORLG00000010663	Chymotrypsin-like elastase family member 2A	*ela3l*	151.4340	19.1180	0.0219	0.1262
ENSORLG00000007210	Transmembrane serine protease 13a	*tmprss13a*	46.9547	11.3090	0.0332	0.2408
ENSORLG00000016693	Deoxyribonuclease-1	*dnase1*	87.5047	19.5877	0.0332	0.2238
ENSORLG00000006967	Chymotrypsin-like elastase family member 1	*CELA1* (1 of many)	1625.2900	386.6150	0.0437	0.2379
ENSORLG00000004534	Chymotrypsin-like elastase family member 2A	*ela2*	3770.4200	779.8280	0.0437	0.2068

*1 *Fragment per kilobase of exon length per million reads*.

*2 *Fold decrease in IL-17A/F1 KO intestine compared to WT*.

**Table 2:** List of genes up-regulated (≥4-fold) in IL-17A/F1 KO medaka intestines.

**Gene_ID**	**Gene name**	**Gene symbol**	FPKM^*1^		***q*-value**	Fold increases^*2^
			**WT**	**IL-17A/F1 KO**		
ENSORLG00000002988	YEATS domain-containing protein 2-like	*YEATS2*	0.0000	20.6734	0.0092	N/A
ENSORLG00000004442	Nuclear receptor subfamily 0, group B, member 2a	*nr0b2a*	60.9465	397.8790	0.0092	6.5283
ENSORLG00000004874	FAM217B	Uncharacterized protein	0.0000	13.4534	0.0092	N/A
ENSORLG00000004922	Ethanolamine-phosphate phospho-lyase	*etnppl*	10.1030	101.4660	0.0092	10.0432
ENSORLG00000005698	Ankyrin repeat domain-containing protein	*ankrd11*	2.6263	30.3002	0.0092	11.5374
ENSORLG00000006223	Solute carrier family 25 member 53	*SLC25A53*	168.2190	840.5860	0.0092	4.9970
ENSORLG00000009024	MAP kinase-interacting serine/threonine kinase 2a	*mknk2a*	12.9887	86.1048	0.0092	6.6292
ENSORLG00000009853	ZPC domain containing protein 5	–	0.0000	8.2575	0.0092	N/A
ENSORLG00000011206	Dachshund protein	dachd	5.1725	32.7172	0.0092	6.3252
ENSORLG00000012249	ZPC domain containing protein 1	si:ch211-14a17.7	0.0000	10.3453	0.0092	N/A
ENSORLG00000014805	Corneodesmosin-like	–	0.0000	15.8826	0.0092	N/A
ENSORLG00000017013	Collagen type I alpha 1	*col1*	6.9284	28.6813	0.0092	4.1397
ENSORLG00000005156	Mitogen-activated protein kinase kinase kinase kinase 4	*MAP4K4* (1 of many)	5.0158	21.7342	0.0234	4.3331
ENSORLG00000001512	Beta, beta-carotene 15,15′-dioxygenase	*bco1l*	116.2480	488.8530	0.0298	4.2053
ENSORLG00000003790	Craniofacial development protein 2-like	*CFDP2*	23.6460	107.9310	0.0360	4.5645

*1 *Fragment per kilobase of exon length per million reads*.

*2 *Fold increase in IL-17A/F1 KO intestine compared to WT*.

### Gene Expression Analysis of Down-Regulated Immune-Related and Digestive Enzyme Genes

**Figure 4:**
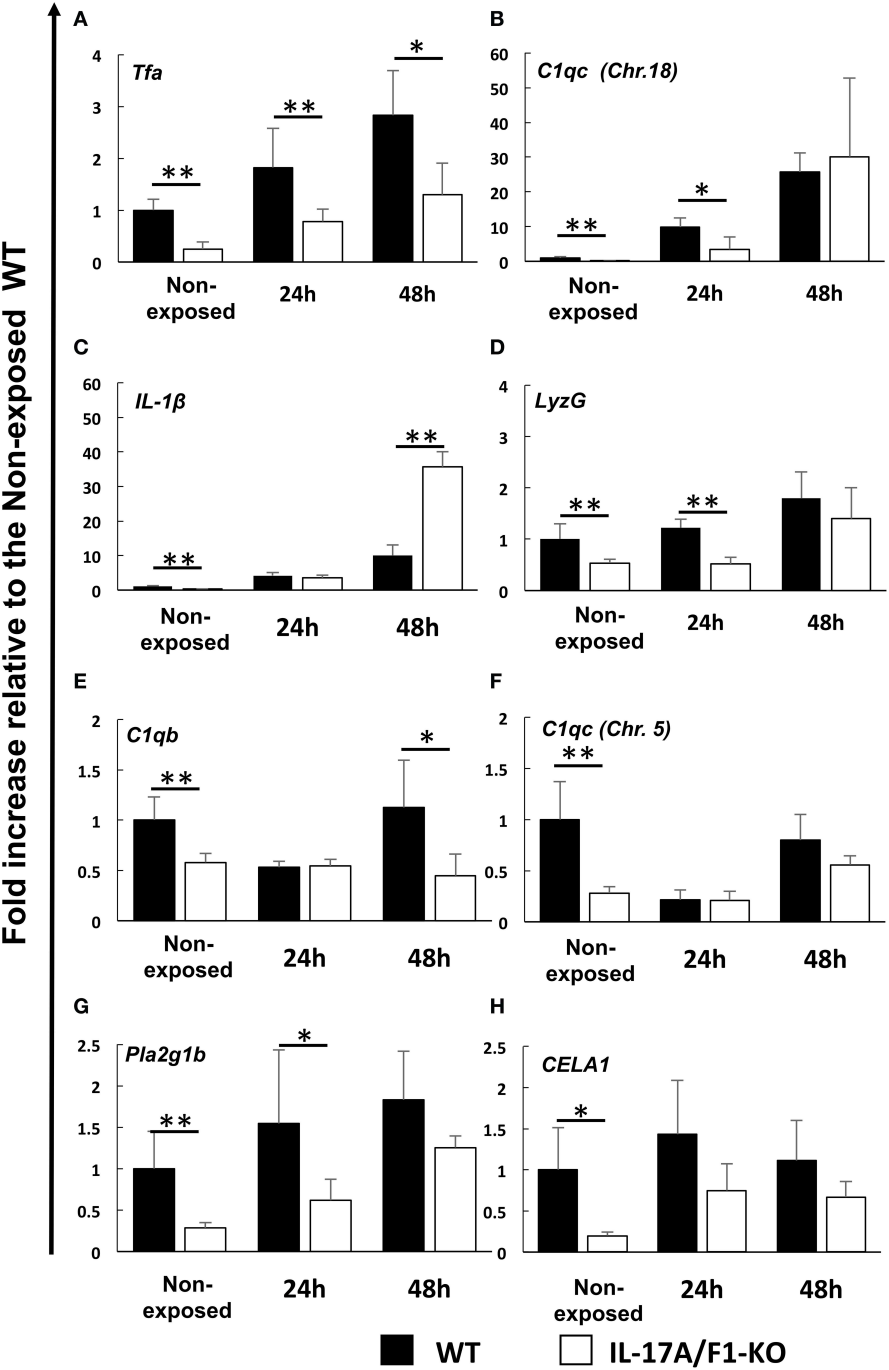
Real-time PCR analysis of the down-regulated immune-related and digestive enzyme genes identified under the healthy condition via RNA-seq. Expression levels of **(A)** transferrin a, **(B)** complement C1q subcomponent subunit C (Chr.18), **(C)** IL-1β, **(D)** G-type lysozyme, **(E)** complement C1q subcomponent subunit b, **(F)** complement C1q subcomponent subunit C (Chr. 5), **(G)** phospholipase A2 (group IB), and **(H)** chymotrypsin-like elastase family member 1 were compared between healthy and exposed conditions (24 and 48 h) of immersed WT and IL-17A/F1 (11-bp insertion strain) medaka in *E. piscicida*-containing tanks (6.9 × 10^8^ CFU/mL). **P < 0.01, *P < 0.05 (Student's *t*-test) WT medaka group vs. KO(+11) medaka group at each time point. Data are presented as mean ± SD; *n* = 5 fish' SD depends on the statistical analyses run.

The genes down-regulated in the IL-17A/F1-KO(-7) intestines were also confirmed in the IL-17A/F1-KO(+11) intestines by qPCR. Furthermore, we analyzed healthy and *E. piscicida*-exposed medaka via qPCR and found that the immune-related genes *Tfa* and *C1qc* (Chr. 18) were significantly reduced (*Tfa*, 0-, 24-, and 48-h exposure; *C1q*, 0- and 24-h exposure) in the intestines of the 11-bp insertion line (Figures 4A,B). Additionally, the proinflammatory cytokine *IL-1*β, antimicrobial peptide G type lysozyme (*LyzG*), and other types of C1q genes [*C1qb* and *C1qc* (Chr. 5)] were found to be significantly decreased in the IL-17A/F1-KO(+11) medaka intestines (*IL-1*β, non-exposed state; *LyzG*, non-exposed state and 24-h exposure; C1qb, non-exposed state and 48-h exposure; C1qc, non-exposed state; Figures 4C–F). Although these genes were down-regulated in the 7-bp deletion line, it was not statistically significant, as evidenced by RNA-seq (Supplementary Table 2). The expression of lipase *pla2g1b* and *CELA1* was also significantly reduced in the IL-17A/F1-KO(+11) (Figures 4G,H). Nevertheless, several immune-related and digestive enzyme genes showed no significant changes between WT and KO (Supplementary Figure 3).

### 16S rRNA Sequencing Quality

We next analyzed the 16S rRNA V3 and V4 amplicon sequences of the bacterial communities from 30 intestinal content samples. WT and IL-17A/F1-KO were compared under non-exposed and *E. piscicida*-exposed conditions. In total, 1,340,455 raw sequence reads were obtained. After adapter and quality trimming, 870,792 reads were used for OTU characterization and OTU-based analysis by QIIME 2. Finally, these sequences were clustered into 2065 OTUs at a 97% identity threshold, with OTUs representing 711 different taxa. For representative OTUs in non-exposed samples, the OTU numbers of KO medaka were higher than for WT (*P* = 0.058). The average number of OTUs was 104 and 137.4 in the WT and KO medaka, respectively. Furthermore, the number of OTUs in the non-exposed WT group was significantly increased compared with that of the two exposed WT groups (non-exposed state, 104.0 OTUs; 24-h exposure, 192.4 OTUs; 48-h exposure, 229 OTUs). However, these significant increases in OTUs after *E. piscicida* exposure were not observed in the KO medaka (Supplementary Figure 4).

### Relative Abundance and Core Bacteria in KO and WT Medaka

**Figure 5:**
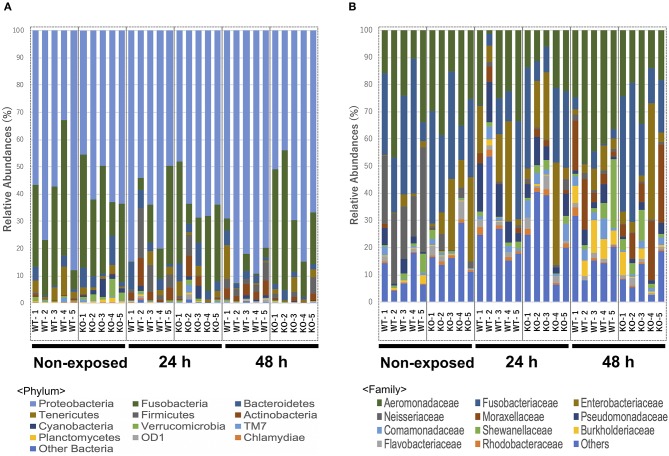
Relative abundance of the intestinal bacterial communities of WT and IL-17A/F1-KO at different taxonomic levels in healthy medaka and medaka immersed for 24 h in *E. piscicida*-containing tanks (2.1 × 10^8^ CFU/mL). **(A)** Relative abundance of gut microbiome communities of WT and IL-17A/F1-KO at the phylum level (*n* = 5). **(B)** Relative abundance of intestinal bacterial communities of WT and IL-17A/F1-KO at the family level. Data were obtained from one experiment with five individual fish (*n* = 5).

**Figure 6:**
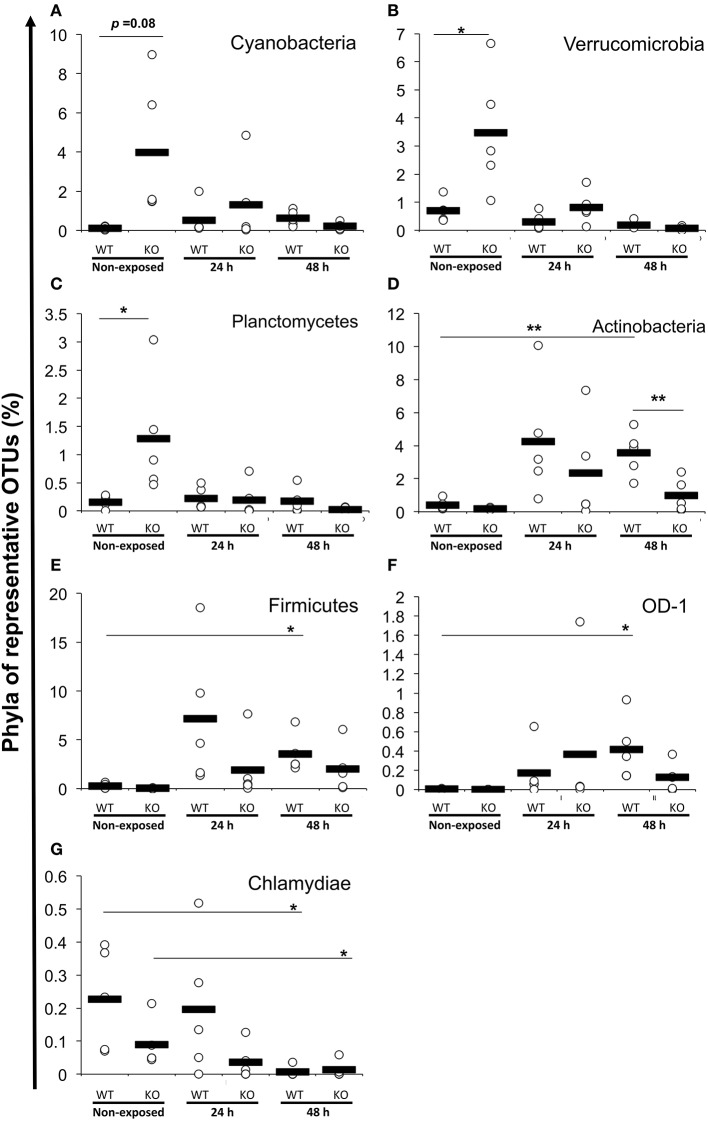
Increased or decreased phylum abundance of the top 12 dominant phylum total reads between WT and KO medaka or against *E. piscicida* exposure. Abundance of **(A)** Cyanobacteria, **(B)** Verrucomicrobia, **(C)** Planctomycetes, **(D)** Actinobacteria, **(E)** Firmicutes, **(F)** OD-1, and **(G)** Chlamydiae. **P < 0.01, *P < 0.05 (Student's *t*-test). Data are from one experiment with five individual fish (*n* = 5).

At the phylum level, Proteobacteria (65.90%) was the dominant phylum, followed by Fusobacteria (20.96%) and Bacteroidetes (3.65%). These phyla of representative OTUs accounted for 90.5% of the reads and were commonly found across all 30 intestinal content samples. Other than these dominant phyla, 31 phyla, including Chlamydiae, OD-1, Planctomycetes, TM7, Verrucomicrobia, Cyanobacteria, Actinobacteria, Firmicutes, and Tenericutes, were identified from the representative OTUs (Figure 5A). At the phylum level, KO medaka showed different intestinal bacterial diversity compared to WT under a non-exposed state. Although not significant, Cyanobacteria showed an increased tendency in the KO compared with WT medaka under a non-exposed state (*t*-test*; P* = 0.08; Figure 6A). The representative OTU percentages of Verrucomicrobia and Planctomycetes were significantly higher in KO than in WT medaka under a non-exposed state (*P* < 0.05; Figures 6B,C). After exposure, WT and KO medaka showed variations in phylum proportions. The proportion of Actinobacteria in the WT increased significantly after 48 h, whereas KO medaka did not show a significant increase. Additionally, Actinobacteria was significantly higher in WT than KO after 48 h exposure (Figure 6D). Furthermore, for Firmicutes and OD-1, WT showed a significant increase after 48 h of exposure, whereas KO did not show a significant increase (Figures 6E,F). Conversely, the Chlamydiae phylum was decreased after 48 h of exposure in both WT and KO (Figure 6G).

At the family level, *Aeromonadaceae, Fusobacteriaceae, Enterobacteriaceae, Neisseriaceae, Moraxellaceae, Pseudomonadaceae, Shewanellaceae, Comamonadaceae, Burkholderiaceae, Flavobacteriaceae*, and *Rhodobacteraceae* were dominant among core bacteria, which occupied over 82% of total reads (Figure 5B). Under a non-exposed state, the proportions of *Enterobacteriaceae, Rhodobacteraceae, Xanthomonadaceae, Cryomorphaceae, OM60*, and *Acetobacteraceae* were significantly higher in KO than WT groups, whereas *Neisseriaceae* and *Pseudomonadaceae* were significantly lower. These significant differences were not observed after 24 h of exposure. However, after 48 h, the WT and KO groups showed significant differences once more in the microbiome at the family level, with increased proportions of *Aeromonadaceae, Neisseriaceae, Corynebacteriaceae, Xanthomonadaceae*, and *Propionibacteriaceae* in the WT compared with the KO group. Meanwhile, only *Fusobacteriaceae* in the KO group was significantly higher than in WT at 48 h (Supplementary Table 3). At the genus level, *Edwardsiella*, for which *E. piscicida* belongs, was detected only in *E. piscicida* immersed groups in both WT and KO groups at 24 and 48 h. However, no significant changes in containing rate were observed between WT and KO (Supplementary Figure 5). Furthermore, at the OTU level, *P. shigelloides* was significantly higher in KO than WT under a non-exposed state (Table 3), which was confirmed by qPCR (Supplementary Figure 6). Other than *P. shigelloides, Cetobacterium somerae* level was significantly higher in KO than WT at 48 h. *Chitinibacter tainanensis*, however, was significantly higher in the WT than the KO group at 0 and 48 h of exposure (Table 3).

**Table 3:** Comparison of gut microbiome OTUs in WT and KO medaka intestines.

	**Average observed OTUs (%)**		***p*-value**
	**WT**	**IL-17A/F1 KO**	
**0 h**			
*Chitinibacter tainanensis*	23.7	2.27	0.0007
*Plesiomonas shigelloides*	1.61	15.29	0.0449
Aeromonadaceae (Family)	1.47	5.05	0.0164
*Pseudomonas* (Genus)	3.13	0.05	0.034
**24 h**			
Pseudomonas (Genus)	4.65	1.33	0.0439
**48 h**			
*Cetobacterium somerae*	2.91	28.87	0.0092
*Chitinibacter tainanensis*	1.39	0.42	0.0035
*Acinetobacter* (Genus)	0.71	5.99	0.0099
Aeromonadaceae (Family)	35.67	20.19	0.0142

### α-Diversity Analysis

**Figure 7:**
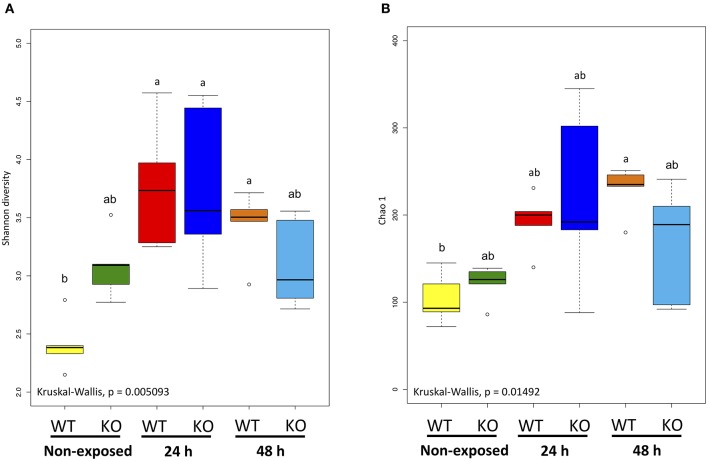
α-Diversity indices of the intestinal bacterial communities of WT and IL-17A/F1-KO medaka. **(A)** Shannon diversity and **(B)** Chao 1 indices were determined by R studio. Different letters above the bars indicate significant differences as determined by Dunn's tests. Data are from one experiment with five individual fish (*n* = 5).

Shannon and Chao 1 diversity indices were determined for α-diversity analysis of the metagenome data. The Shannon diversity index indicated that bacterial diversity in the WT increased significantly as exposure progressed (non-exposed state vs. 24-h exposure, *P* < 0.01; non-exposed state vs. 48-h exposure, *P* < 0.05). However, between the KO groups, no significant changes were observed (Figure 7A). The Chao 1 diversity index showed a significant change (*P* < 0.05) only in the WT after 48 h exposure when compared to non-exposure (Figure 7B).

### Weighted UniFrac Distance-Based Analysis

**Figure 8:**
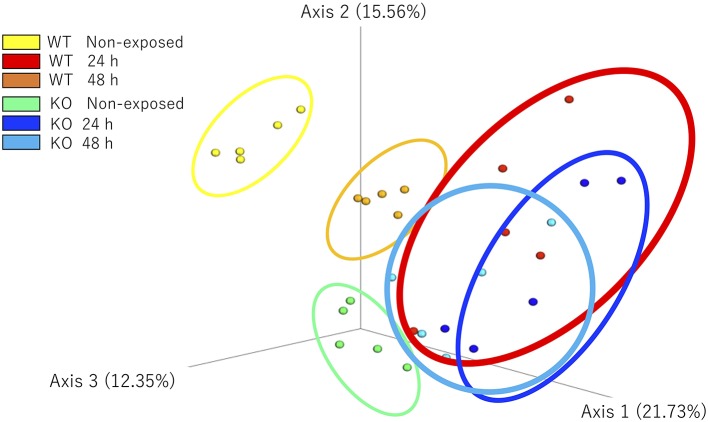
β-Diversity indices of the intestinal bacterial communities of WT and IL-17A/F1-KO medaka. Weighted UniFrac distance-based analyses were performed by R studio. Different letters above the bars indicate significant differences as determined by PERMANOVA. Data are from one experiment with five individual fish (*n* = 5).

The microbial community composition of the WT and KO as well as each non-exposed and *E. piscicida*-exposed groups were visualized according to the weighted UniFrac distance-based analysis (with statistical confirmation), which accounts for bacterial abundance rather than presence and absence of taxa. In non-exposed samples, WT and KO groups formed significantly different clusters (*P* = 0.011). After 24 h of exposure, these clusters became mixed and showed no significant differences. However, after 48 h of exposure, the WT and KO groups formed different clusters again (*P* = 0.018). Comparisons between the exposed and non-exposed WT groups showed that the non-exposed groups at 24 and 48 h were in statistically different clusters (non-exposed state vs. 24 h, *P* = 0.011; non-exposed state vs. 48 h, *P* = 0.007; 24 h vs. 48 h, *P* = 0.009). However, in the KO groups, no significant differences were observed between 24 and 48 h of exposure (non-exposed vs. 24 h, *P* = 0.013; non-exposed state vs. 48 h, *P* = 0.005; 24 h vs. 48 h, *P* = 0.122; Figure 8).

## Discussion

In this study, we established two strains of IL-17A/F1-KO medaka. Medaka IL-17A/F1 and 2 are adjacent on the same chromosome as well as mammalian IL-17A and -F, whereas IL-17A/F3 is located on a different chromosome (20). Gene expression analysis of IL-17A/F1, 2, and 3 in the medaka intestinal tract revealed that IL-17A/F3 levels were the highest, whereas IL-17A/F2 levels were extremely low. This expression pattern of IL-17A/F1, 2, and 3 was previously reported in yellow croaker (*Larimichthys polyactis*) (23). In contrast, IL-17A/F2 expression in the intestinal tract of salmonids was relatively high (22). Thus, IL-17A/F1, 2, and 3 gene expression levels are different among fish species. In teleost IL-17A/F1, it was reported that yellow croaker IL-17A/F1 has a higher ability to activate the transcriptional activity of NF-κB than IL-17A/F2 and 3 (23). Furthermore, in Japanese pufferfish, IL-17A/F1 showed a more acute up-regulated response compared to IL-17A/F3 (20). Based on these previous studies, we selected medaka IL-17A/F1 as a target gene of genome editing. However, the differences in the functionality of IL-17A/F1, 2, and 3 remain unclear, which warrants future research to increase our understanding of these genes.

In mammalian small intestines, IL-17A and F play important roles in the production of AMPs, such as defensin and calprotectin (11, 14). In the present study, we performed transcriptome analysis (RNA-seq) to compare gene expression pattern between WT and IL-17A/F1-KO intestines to elucidate the role of medaka IL-17A/F1. Expression levels of immune-related genes, such as *Tfa* and *C1qc*, and various lipases and proteinases, such as phospholipase A2 and chymotrypsin, were found to be down-regulated in the intestines of IL-17A/F1-KO medaka compared to WT. The induction of these genes has not been reported to depend on IL-17A and F in mammals.

Among the genes with reduced expression in KO individuals, several have been reported to be important in regulating the gut microbiome in vertebrates. Transferrin a is an iron ion transporter and an important factor in homeostasis maintenance, in which the iron ion absorption effect is known to attenuate bacterial proliferation. Transferrin a is also abundant in mucosal tissues and considered to inhibit bacterial survival (32, 33). Moreover, the transferrin gene has been identified in several fish species, including the Japanese medaka, and is known to be induced at the transcriptional level upon bacterial infection stress (34–36). C1q is known to trigger initiation of the classical pathway of the complement system (37). In zebrafish, C1q production via the transcription factor interferon regulatory factor-8 (IRF8) in macrophages modulates the intestinal microbiome (38).

Digestive enzymes are also important for regulation of the intestinal tract microbiome and immune-related genes, such as *Tfa* and *C1q*. Members of the chymotrypsin and elastase family are enzymes involved in the degradation of the outer membrane protein OmpA of Gram-negative bacteria (39). Although phospholipase A2 (group IB; *pla2g1b*) mainly acts as a lipid degrading enzyme, phospholipase A2 (*PLA2*) produced from intestinal epithelial cells is also important for defense against parasite infection (40). Furthermore, phospholipase A2 (group IIA; *pla2g2A*), which is classified as the same phospholipase, is known as a marker of Paneth cells. In fact, secreted *PLA2* modulates intestinal stem cell and Paneth cell differentiation (41). In addition, expression levels of *pla2g2A* in IL-17A- or F-KO mouse intestines are reduced compared with those of WT mice, with IL-17F-KO mice showing a particularly marked decrease (42). Thus, the significant down-regulation of several genes, as shown by RNA-seq, appear to affect gut microbiome control in the intestinal tract of IL-17A/F1-KO(-7) medaka. Considering that the expression of IL-17 KO-dependent genes was different from that in mammals, we theorized that the fish intestinal tract may have a unique and specific IL-17A/F1-mediated microbiome control mechanism, which differs from that of the mammalian intestinal tract.

Gene expression analysis by qPCR confirmed that the two IL-17A/F1 mutant strains established in this study showed similar trends in gene down-regulation; *Tfa, C1qc, C1qb, IL-1*β, *lyzG*, and the lipase and proteinase genes *PLA2* and *CELA1* were down-regulated compared to the WT strain. RNA-seq results showed that, although not significant, expression levels of *IL-1*β*, LyzG, and C1q* genes [*C1qb* and *C1qc* (Chr. 5)] were decreased in the IL-17A/F1 knockout. In mammalian intestines, lysozyme is known as a marker of Paneth cells, which secrete AMPs from the intestinal epithelia layer (43). In this study, RNA-seq indicated that expression levels of both lysozymes, *LyzC* and *lyzG*, were reduced. However, *LyzC* expression was not significantly decreased in the qPCR analysis. It is of note that expression levels of Paneth cell markers (e.g., *PLA2* and *LyzC*) were reduced in the IL-17A/F1-KO medaka strains, although Paneth cells have not yet been identified in fish. Moreover, among the down-regulated genes identified by RNA-seq, *IL-1*β was the only gene induced via mammalian IL-17 signaling. In the mammalian intestinal tract, IL-17A and F stimulate macrophages to induce IL-1β and promote the aggregation of neutrophils and other lymphocytes (12). In addition, IL-1β is essential for the differentiation of lymphocytes, including Th17 and γδ T cells, which are IL-17-producing cells (44). Moreover, in fish, it has been reported that recombinant IL-17A/F1 of grass carp (*Ctenopharyngodon Idella*) and IL-17A/F2 of common carp (*Cyprinus carpio*) induce IL-1β *in vitro* (45, 46). However, it has not been reported that IL-1β is induced in an IL-17A/F-dependent manner in the fish intestinal tract.

For metagenomic analysis, the IL-17A/F1-KO(+11) strain was used, and changes in the gut microbiome during *E. piscicida* exposure were analyzed. Between WT and KO groups, there was no remarkable difference in the number of OTUs or diversity. Under a non-exposed state, however, WT and IL-17A/F1-KO medaka exhibited different gut microbiomes. The observed number of OTUs and the α-diversity index increased in the KO strain compared with the WT. Furthermore, the β-diversity test revealed that the two groups were in significantly different clusters. In a previous β-diversity test, IL-17A-KO mice formed a different gut microbiome to WT mice (47), with IL-17F-KO mice showing similar results (42). In addition, mice with knock out of IL-17RA and RC, which are receptors for IL-17A and F, respectively, are highly sensitive to the immune disease, Graft-vs.-host disease (GVDH). Moreover, after co-housing WT mice with IL-17RA- or RC-KO mice, the gut microbiome of WT mice became similar to that of IL-17RA/RC-KO mice, with increased sensitivity to GVHD (48). Based on these findings, the health importance of gut microbiome regulation by IL-17A and F signaling via IL-17RA/RC in mammals is clear. However, this importance has not been shown in fish. Our findings suggest that the IL-17A/F gene is an important inflammatory cytokine for regulating the gut microbiome in fish. For a better understanding of this role of IL-17A/F, it is necessary to simultaneously analyze other IL-17A/F ligands (i.e., IL-17A/F2 and/or 3) and their receptors (i.e., IL-17RA and RC).

Prior to metagenomic analysis, we compared the susceptibility of WT and IL-17A/F1-KO(+11) strains to *E. piscicida* exposure, but no differences in cumulative mortality after exposure were observed (Supplementary Figure 7). The effect of IL-17A/F1-KO on the gut microbiome may be limited to the control of opportunistic bacteria resident in the intestines. Opportunistic bacteria were examined at the phylum level, and the results revealed Proteobacteria as the most dominant phylum in the intestinal microbiome of medaka, followed by Fusobacteria. By contrast, the gut microbiome of mammals at the phylum level is dominated by Firmicutes and Bacteroides, with only a low percentage of Proteobacteria (49). However, the fish gut microbiome has been reported to be dominated by Proteobacteria and Fusobacteria, which is consistent with our results. In addition, in both freshwater and marine fish species, the predominance of Proteobacteria and Fusobacteria is common (50, 51); however, this is not a simple reflection of the bacteria present in the surrounding aquatic environment. Shrimp, which is also an aquatic organism, has predominant Proteobacteria levels but hardly contains Fusobacterium (52), whereas the whale gut microbiome is similar to that of land mammals (53). Although the percentage of Proteobacteria and Fusobacteria in this study showed no significant change between the WT and KO strains, others types of phyla showed different composition rates between WT and KO. Under a non-exposed state, Cyanobacteria, Verrucomicrobia, and Planctomycetes were increased in the KO gut microbiome compared with the WT. Cyanobacteria are known to play important roles in the activation of host cell signaling by producing their primary metabolites, but the cyanotoxins produced may be harmful to many vertebrate species (54). Meanwhile, at the OTU level, significant changes were observed between WT and KO with regards to several bacterial species. No pathogenicity has been reported for any of these species, except for *P. shigelloides*. *P. shigelloides* is an intestinal bacterium that is widely known as a causative agent of food poisoning in humans and is also present in freshwater. In recent years, *P. shigelloides* infection has been reported to be responsible for the high mortality of several freshwater fish species as it is an opportunistic pathogen (55). Increases in the proportion of *P. shigelloides* in the KO strains may indicate an increased risk of death due to an opportunistic infection. The effects of *P. shigelloides* on health and changes in susceptibility to infections other than *E. piscicida* have not been clarified in this study, warranting future research.

In summary, we established IL-17A/F1-deficient Japanese medaka strains in this study. In the IL-17A/F1-KO medaka intestines, the expression of various immune-related genes as well as proteinase and lipase genes was significantly down-regulated. Furthermore, 16S rRNA-based metagenomic analysis revealed that the WT and KO strains formed different gut microbiomes, with a significantly increased proportion of *P. shigelloides—*a fish opportunistic pathogen—in the KO strains, suggesting that medaka IL-17A/F1 may have a role in maintaining healthy intestines by modulating the gut microbiome via transcriptional control of the affected genes.

## Data Availability Statement

The datasets generated for this study can be found in the DDBJ Sequence Read Archive (DRA) under accession number DRA008715, DRA008844, GenBank accession number AJ300545.

## Ethics Statement

The animal study was reviewed and approved by University of Miyazaki.

## Author Contributions

YO, TA, MS, and JH designed the research. YO, NMo, DI, NMi, HM, MK, TK, and JH conducted the research. YO, DI, SW, YS, HT, and TK analyzed the data. YO, MS, and JH wrote the paper. All authors reviewed and approved the final manuscript.

### Conflict of Interest

The authors declare that the research was conducted in the absence of any commercial or financial relationships that could be construed as a potential conflict of interest.
