# Supplementary material for: Interleukin-17A/F1 Deficiency Reduces Antimicrobial Gene Expression and Contributes to Microbiome Alterations in Intestines of Japanese medaka (Oryzias latipes)
Source: Front Immunol. 2020 Mar 17;11:425. doi: 10.3389/fimmu.2020.00425 (PMC7092794; doi:10.3389/fimmu.2020.00425)
Supplement: Supplementary file 1 [file Data_Sheet_1.PDF]

Supplementary materials

Article information:

IL-17A/F1 Deficiency Reduces Antimicrobial Gene Expression and Contributes to Microbiome Alterations in Intestines of Japanese medaka (*Oryzias latipes*)

Yo Okamura[1], Natsuki Morimoto[1], Daisuke Ikeda[2], Nanami Mizusawa[2], Shugo Watabe[2], Hiroshi Miyanishi[3], Yuichi Saeki[4], Haruko Takeyama[5], Takashi Aoki[6], Masato Kinoshita[7], Tomoya Kono[4], Masahiro Sakai[4] Jun-ichi Hikima[4]*

[1] Interdisciplinary Graduate School of Agriculture and Engineering, University of Miyazaki, Miyazaki 889-2192, Japan
[2] School of Marine Biosciences, Kitasato University, Sagamihara, Kanagawa 252-0373, Japan
[3] Department of Marine Biology and Environmental Science, Faculty of Agriculture, University of Miyazaki, Miyazaki 889-2192, Japan
[4] Department of Biochemistry and Applied Bioscience, Faculty of Agriculture, University of Miyazaki, Miyazaki 889-2192, Japan
[5] Department of Life Science & Medical Bioscience, School of Advanced Science and Engineering, Waseda University, Shinjuku-ku, Tokyo, 169-8555, Japan
[6] Research Organization for Nano and Life Innovation, Waseda University, Tokyo, Japan
[7] Division of Applied Bioscience, Graduate School of Agriculture, Kyoto University, Kitashirakawa-Oiwake-cho, Sakyo-ku, Kyoto, 606-8502, Japan

Running title: [IL-17A/F1 deficiency in Japanese medaka]

List of supplementary materials:

Supplementary Table 1. The nucleotide sequences of primers and crRNAs used in this study

Supplementary Table 2. List of the other immune-related genes showing down-regulation tendency in IL-17A/F1-KO intestine by RNA-seq

Supplementary Table 3. Comparison of intestinal bacteria of WT and KO groups at the family level

Supplementary Figure 1. Efficiency of mutation by each crRNA and HMA results of the mutated medaka.

Supplementary Figure 2. No appearance changes were observed between WT and KO intestinal tracts.

Supplementary Figure 3. Quantitative real-time PCR analysis of down-regulated immune-related and digestive enzyme genes in the healthy condition of medaka intestines.

Supplementary Figure 4. Total number of observed OTUs in WT and KO medaka.

Supplementary Figure 5. The abundance of *Edardsiella* in WT and KO medaka after *E. piscicida* exposure.

Supplementary Figure 6. Quantitative bacterial copy numbers of *Plesiomonas shigelloides* calculated by the plasmid copies number method using qPCR in healthy and immersed medaka in the tank containing *E. piscicida* (2.1×10^{8} CFU/mL).

Supplementary Figure 7. Survival rate against *E. piscicida* exposure in WT and IL-17A/F1-KO medaka.

Suppl. Table. 1

Supplementary Table 1. The nucleotide sequences of primers and crRNAs used in this study

Primer name	Nucleotide sequence (5'-3')	Amplicon length	Purposes
crRNA 1	GAUGAUGAUGAUGAGGAUGAUGG		Genome editing
crRNA 2	CCGAGGCGGCAGCCGUGCCGAAG		Genome editing
crRNA 3	CCGUGCCGAAGGCGUCAAAGACG		Genome editing
IL-17aF1	GCTCCTCTGGCCTTGATGATG	171 bp (WT)	HMA
IL-17aR1	CTTGTACGTCCACGGCGAGATG		
Ol_IL-17A/F1-exF1	TTCAGCAACCAGCTTCTGC	91 bp	qPCR
Ol_IL-17A/F1-exR1	GGTCACCATCATCCTCATCA		
Ol_IL-17A/F2-exF5	ACCCAAGTGACAGGATGGAG	120 bp	qPCR
Ol_Il-17A/F2-exR6	CATTGCAGTCAGGAGGATGA		
Ol_IL-17A/F3-exF1	GCTTCTGGTTCTGAGAGCTT	101 bp	qPCR
Ol_IL-17A/F3-exR1	ACTTTGGTCCTTGCTTCAGC		
Ol_Tfa-exF1	GACGGTAACGGTCCTGATTG	139 bp	qPCR
Ol_Tfa-exR1	GAGTCACTACTGCGTGTGCT		
Ol_lyzG-exF1	GTGTCAGGTGTGAGTGCATC	134 bp	qPCR
Ol_lyzG-exR1	CTGGAGATGATGCCGGCAAT		
Ol_lyzC-exF1	GTGTTTGAGCGCTGTCAGTG	239 bp	qPCR
Ol_lyzC-exR1	GCGCTGCATGGAATTTTGCA		
Ol_C1qc (Chr. 18)-exF1	AGGAATCTTCACTGCGCCTG	145 bp	qPCR
Ol_C1qc (Chr.18)-exR1	TCCATCGTGTGATGTCTGGT		
Ol_C1qc (Chr. 5)-exF1	ATGCCAGGTATTCCTGGCCTT	146 bp	qPCR
Ol_C1qc (Chr.5)-exR1	TGACCTCGCTTACCAGGTGATC		
Ol_C1qb-exF1	CAGTATTATGGCCTGTCAGAGGC	150 bp	qPCR
Ol_C1qb-exR1	TTGCCGTTTGGTCCATGGGTT		
Ol_pglyrp2-exF1	GAAACTGACCCTGGCTGTTC	117 bp	qPCR
Ol_pglyrp2-exR1	TCAGGGTTCTCATCCTCCAC		
Ol_Elastase1-exF1	GCAGGGTACGACATTGCTCT	132 bp	qPCR
Ol_Elastase1-exR1	GCCCCATCCACTAATGTAGC		
Ol_Elastase2-exF1	CTCACTGCTGCTCACTGCAT	123 bp	qPCR
Ol_Elastase2-exR1	CTCGTGGACGATGATCCTTT		
Ol_CPA1-exF2	CCCCTGAATGTGCTCAAGTT	94 bp	qPCR
Ol_CPA1-exR2	CCTGAGTGACCCATTCCCTA		
Ol_Bile salt-activated lipase-exF1	TCCATCTGTCTGGCTCTCCT	102 bp	qPCR
Ol_Bile salt-activated lipase-exR1	GGAACAAGTTGGAAGGCTCA		
Ol_PLA2-exF1	GCTGTGGGTCTTTCTGTGGT	138 bp	qPCR
Ol_PLA2-exR1	GCCTCCAAGTCCACAGTAGC		
Ol_IL-1b-exF	GTCCAGCTGAACATGTCTAC	116 bp	qPCR
Ol_IL-1b-exR	TTGTCTCCTTCTTGGTGGCA		
Ol_ β-actin-exF1	CCACCATGTACCCTGGAATC	153 bp	qPCR
Ol_ β-actin-exR1	GCTGGAAGGTGGACAGAGAG		
V3V4f_MIX	ACACTCTTTCCCTACACGACGCTCTTCCGATCT-NNNNN-CCTACGGGNGGCWGCAG		Metagenomic analysis
V3V4r_MIX	GTGACTGGAGTTCAGACGTGTGCTCTTCCGATCT-NNNNN-GACTACHVGGGTATCTAATCC		
2nd-F	AATGATACGGCGACCACCGAGATCTACAC-XXXXXXXX(*1)-ACACTCTTTCCCTACACGACC		Metagenomic analysis
2nd-R	CAAGCAGAAGACGGCATACGAGAT-XXXXXXXX(*1)-GTGACTGGAGTTCAGACGTGTG		

*1 Index sequences

Suppl. Table. 2

Supplementary Table 2. List of the other immune-related genes showing down-regulation tendency in the intestine of IL-17A/F1-KO medaka

Gene_ID	Gene name	Gene symbol	FPKM*1		*P*-value	Fold decrease*2
			WT	IL-17A/F1 KO		
ENSORLG00000000217	Interleukin-1β	IL-1β	40.5411	11.5106	0.6254	0.283924215
ENSORLG00000000313	Goose-type lysozyme	*lyzG*	147.524	41.9876	0.07254	0.284615385
ENSORLG00000015344	Chicken-type lysozyme	*lyzC*	12.2274	5.28191	0.98992	0.431973273
ENSORLG00000017261	Complement C1q subcomponent subunit B	*c1qb*	172.684	68.5888	0.18583	0.39719256
ENSORLG00000017257	Complement C1q subcomponent subunit C (Chr. 5)	*c1qc*	140.639	63.3303	0.43436	0.45030397

*1 Fragment per kilobase of exon length per million reads
*2 Fold decrease in IL-17A/F1-KO intestine comparing to those of WT

Suppl. Table. 3

Supplementary Table 3. Comparison of intestinal bacteria of WT and KO groups at the family level

Bacterial family name	WT1	WT2	WT3	WT4	WT5	WT average	KO1	KO2	KO3	KO4	KO5	KO average	*P*-value
0 h (non-infection)													
Enterobacteriaceae	0.32	0.29	4.56	0.90	2.06	1.63	1.07	7.92	26.17	10.20	31.10	15.29	0.04507
Neisseriaceae	24.85	25.71	19.15	15.03	38.54	24.66	0.64	6.26	0.47	3.71	2.36	2.69	0.00070
Pseudomonadaceae	6.62	0.67	5.45	2.49	0.62	3.17	0.20	0.24	0.06	0.10	0.07	0.14	0.03892
Rhodobacteraceae	0.50	0.16	0.20	0.29	0.13	0.26	0.64	1.23	1.03	1.42	0.53	0.97	0.00435
Xanthomonadaceae	0.00	0.00	0.00	0.10	0.05	0.03	1.21	1.54	1.54	3.26	1.34	1.78	0.00167
Cryomorphaceae	0.02	0.01	0.00	0.03	0.13	0.04	0.77	0.89	0.19	1.31	0.05	0.64	0.03238
OM60	0.05	0.00	0.04	0.04	0.00	0.03	0.23	0.63	0.86	1.68	0.61	0.80	0.01207
Acetobacteraceae	0.15	0.05	0.00	0.09	0.08	0.07	0.35	0.37	0.28	0.61	0.16	0.35	0.00704
24 h (*E. piscicida*-infection)													
No Family significant Changes showed between WT and IL-17A/F1-KO groups in 24 h.													
48 h (*E. piscicida*-infection)													
Aeromonadaceae	24.63	48.99	44.70	41.75	36.39	39.29	24.40	19.20	34.63	13.88	18.37	22.10	0.01408
Fusobacteriaceae	4.58	0.19	6.31	0.76	3.39	3.04	42.23	51.30	18.97	12.97	19.13	28.92	0.00927
Neisseriaceae	2.11	2.17	1.10	1.32	1.08	1.56	0.32	0.28	0.59	0.59	0.97	0.55	0.00593
Corynebacteriaceae	1.49	0.73	1.12	0.78	2.33	1.29	0.08	0.05	0.61	0.03	0.76	0.31	0.01859
Xanthomonadaceae	0.16	0.12	0.11	0.11	0.08	0.11	0.05	0.00	0.02	0.00	0.08	0.03	0.00472
Propionibacteriaceae	0.67	0.25	0.60	0.50	0.90	0.58	0.13	0.00	0.37	0.04	0.24	0.16	0.00926

Suppl. Fig. 1

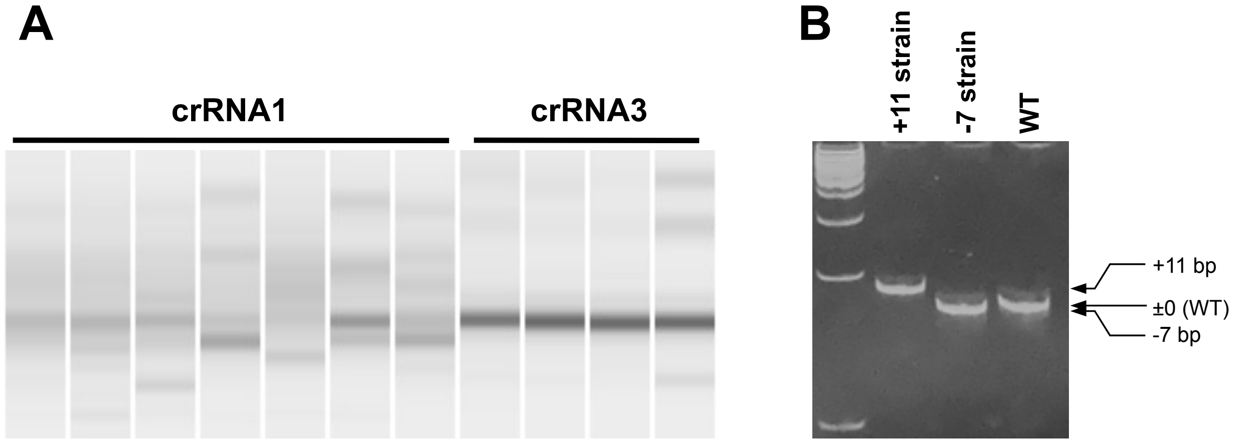

Supplementary Figure 1. Efficiency of mutation by each crRNA and HMA results of the mutated medaka. (**A**) The results of HMA examined for the fertilized medaka eggs treated by crRNA1 or crRNA3 are shown. (**B**) The results of HMA examined for the mutated medaka, 7 bp-deleted (derived from crRNA1) and 11 bp-inserted strains (derived from crRNA3) are shown on 3% agarose gel.

Suppl. Fig. 2

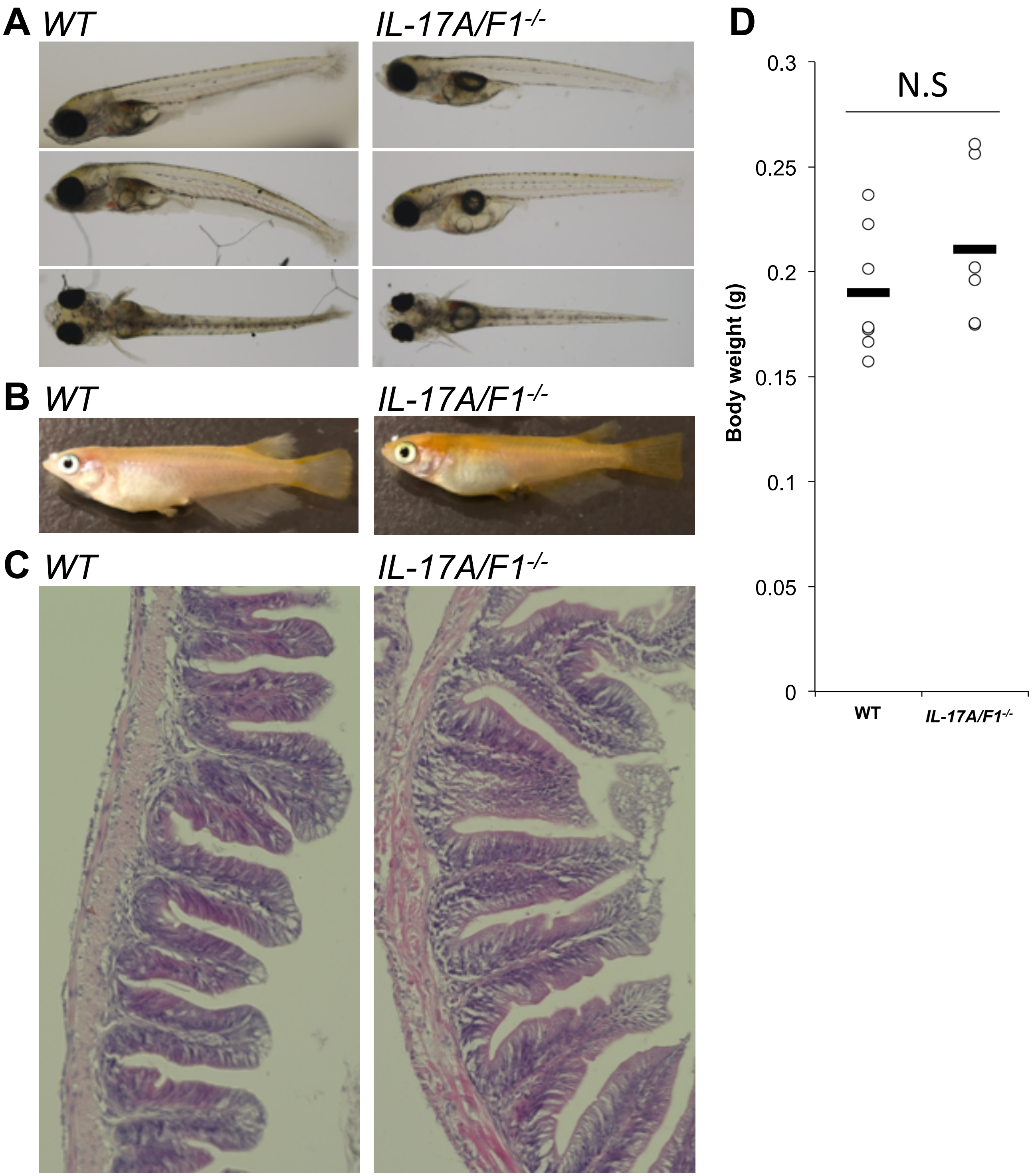

Supplementary Figure 2. No apparent differences in appearance were observed between WT and KO villus tissues of the intestinal tracts. (**A, B**) Comparison of WT and KO. In KO strain (+11 bp inserted strain), both stages in fry of 1 dpf in **A** and 4-month-old adult fish in **B** showed no significant changes. (**C**) Hematoxylin and Eosin (HE) staining in posterior intestine. Histological changes of Intestinal villus tissue were not observed in intestinal tract of KO (+11 bp inserted strain). The observed villus tissues of intestinal tract were sampled from anterior section of the intestine in 4-month-old adult fish. After isolating, intestinal tissues were fixed in 4% PFA/0.1 M PB solution and embedded in paraffin. Sections (5μm) were stained with hematoxylin-eosin for microscopic observation (20× resolution).(**D**) Body weight of WT and KO adult fish (4-month-old). In WT and KO group, no significant changes were observed. Body weight were measured after anesthetizing medaka using MS-222 and wiped off the water of the body (n = 7).

Suppl. Fig. 3

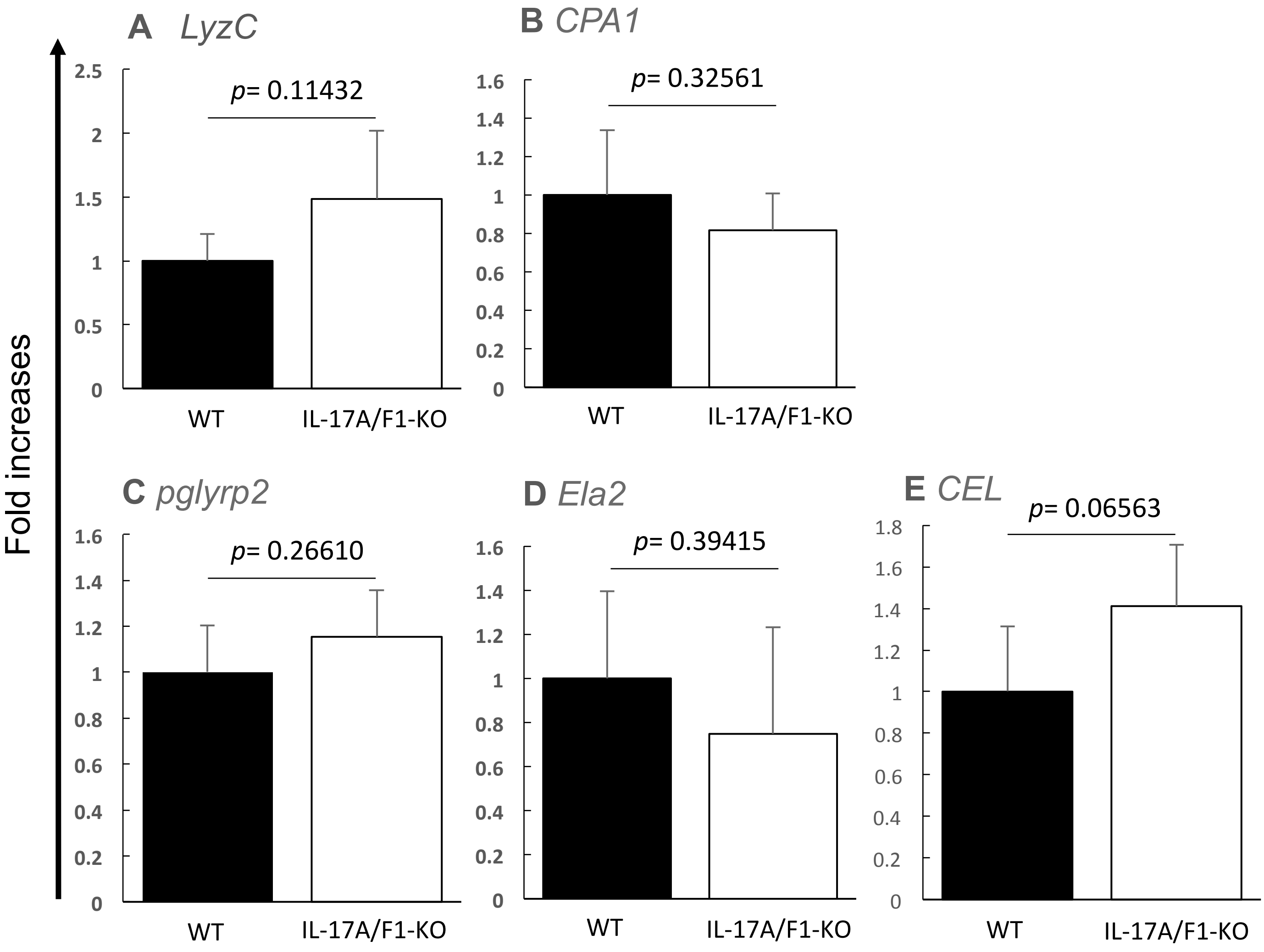

Supplementary Figure 3. Quantitative real-time PCR analysis of down-regulated immune-related and digestive enzyme genes under the healthy condition of medaka intestines. qPCR did not detect down-regulation of these immune related and digestive enzyme genes in the intestine of IL-17A/F1 KO (11 bp inserted strain). The $2^{-\triangle\triangle Ct}$ method was used to calculate and normalize mRNA expression levels using the *β*-actin gene as the internal standard. Each *p*-value was calculated by Student's *t*-test. These comparison were performed only in healthy medaka. Asterisk above bar indicates a significant difference (**; $p < 0.01$, *; $p < 0.05$) by Student's *t*-test in expression levels between the healthy medaka group and unstimulated control medaka group at each time point. (**A**) *C-type lysozyme* (*LyzC*), (**B**) *Carboxypeptidase A1* (*CPA1*), (**C**) *Peptidoglycan recognition protein 2* (*pglyrp2*), (**D**) *Chymotrypsin-like elastase family member 2A* (*Ela2*), (**E**) *Bile salt-activated lipase* (*CEL*). Data are from one experiment with five individual medaka (n = 5).

Suppl. Fig. 4

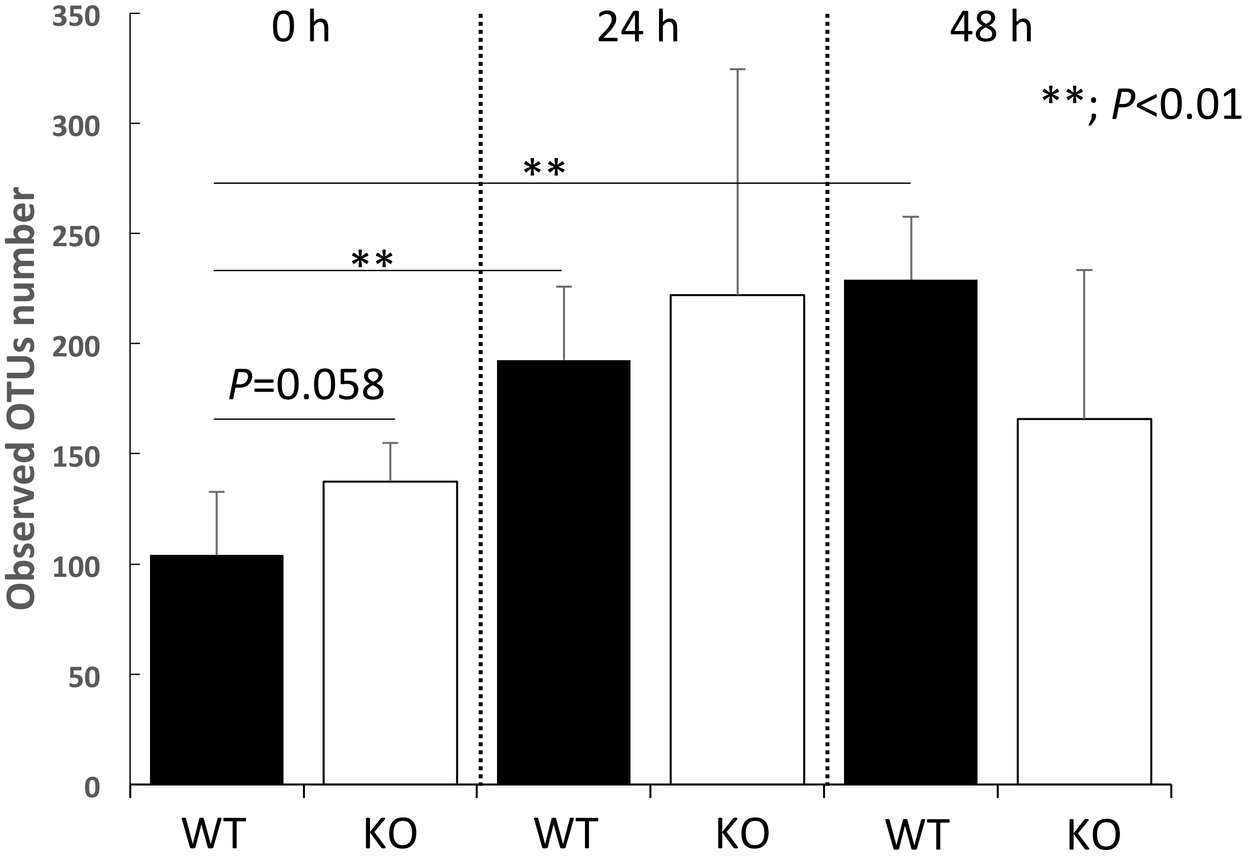

Supplementary Figure 4. Total number of observed OTUs in WT and KO medaka. Total number of observed OTUs in each individual sample was compared between WT and IL-17A/F1-KO in healthy medaka and 24 h-immersed medaka in the tank containing *E. piscicida* ($2.1×10^8$ CFU/mL). Data were obtained from one experiment with five individuals (n = 5). The qualified reads were clustered to generate operational taxonomic units (OTUs) at the 97% similarity level.

Suppl. Fig. 5

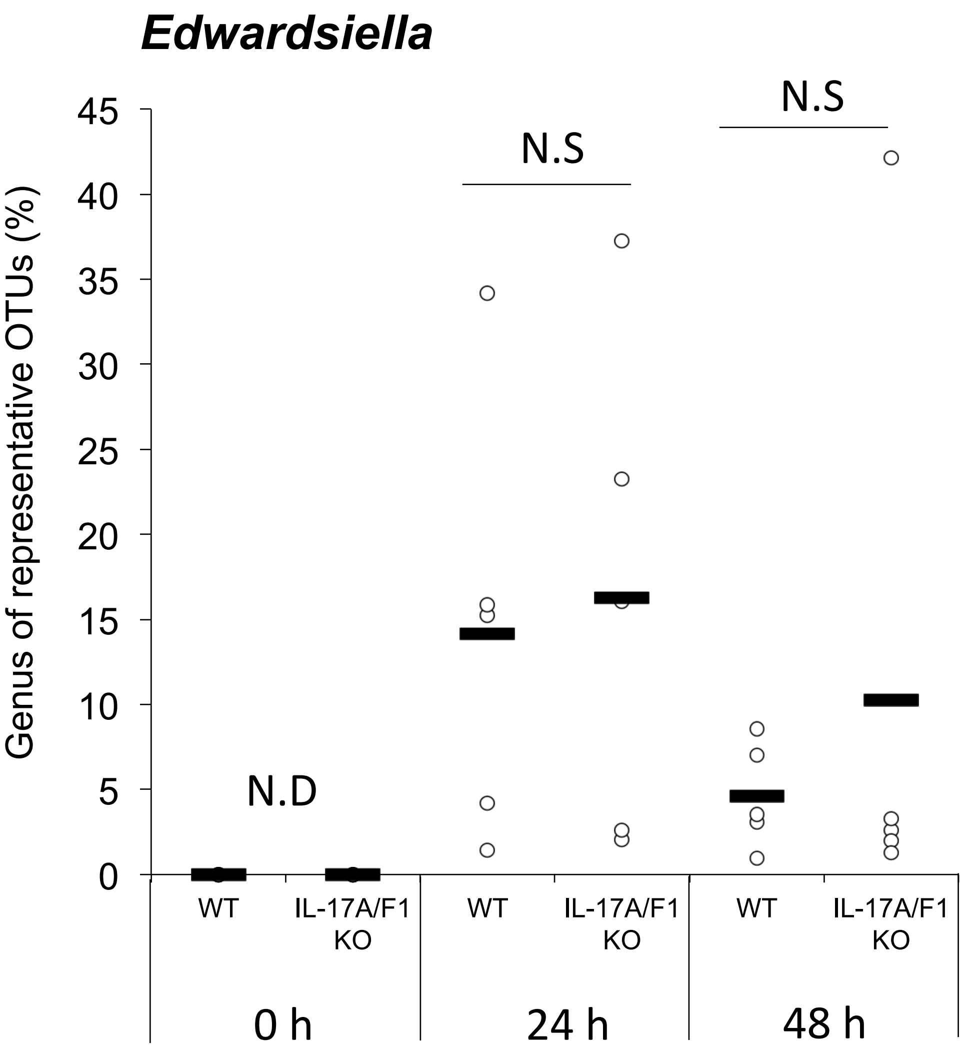

Supplementary Figure 5. The abundance of *Edardsiella* in WT and KO medaka after *E. piscicida* exposure. In the genus level, the *Edwardsiella,* which *E. piscicida* belongs was detected only *E. piscicida* immersed groups in both of the WT and KO in 24 and 48 h. However no significant changes of these containing rate was observed between WT and KO. Data are from one experiment with five individual fish (n = 5).

Suppl. Fig. 6

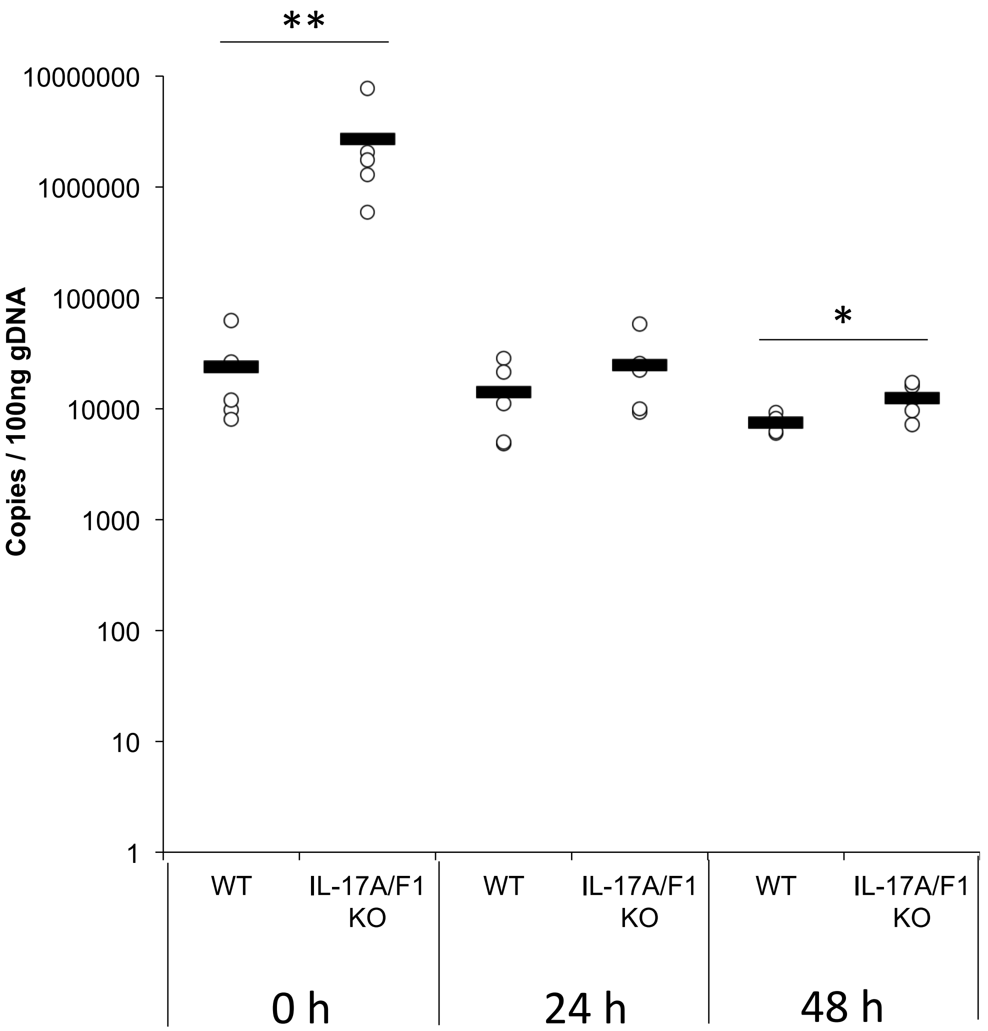

Supplementary Figure 6. Quantitative bacterial copy numbers of *Plesiomonas shigelloides* calculated by the plasmid copies number method using qPCR in healthy and immersed medaka in the tank containing *E. piscicida* (2.1×10^8 CFU/mL). Data were obtained from one experiment with five individuals (n = 5). Asterisks above bars indicate a significant difference (**; $p < 0.01$, *; $p < 0.05$) by Mann-Whitney U test.

Suppl. Fig. 7

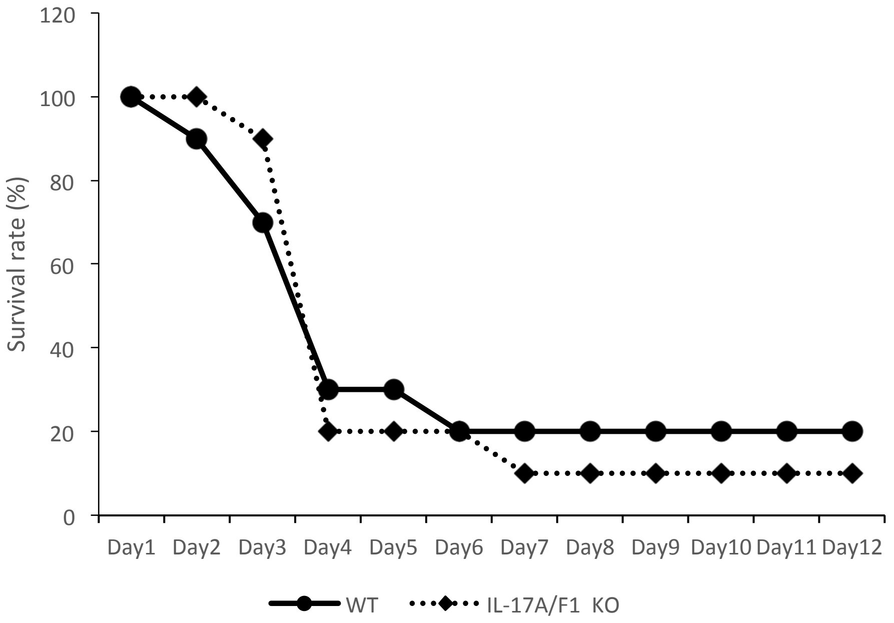

Supplementary Figure 7. Survival rate against *E. piscicida* exposure in WT and IL-17A/F1-KO medaka (n = 10). Mortality of IL-17A/F1-KO(+11) was confirmed by WT against *E. piscicida* (E381 strain). Immersion infection was performed in 10 L water tank set up at 28 ℃, and bacterial concentration of each tank was set up to 2.9×10^7 CFU/mL. After starting immersion, bacterial solution was replaced with 20% fresh water daily.
